# Artificial intelligence-enabled fully automated detection of cardiac amyloidosis using electrocardiograms and echocardiograms

**DOI:** 10.1038/s41467-021-22877-8

**Published:** 2021-05-11

**Authors:** Shinichi Goto, Keitaro Mahara, Lauren Beussink-Nelson, Hidehiko Ikura, Yoshinori Katsumata, Jin Endo, Hanna K. Gaggin, Sanjiv J. Shah, Yuji Itabashi, Calum A. MacRae, Rahul C. Deo

**Affiliations:** 1 One Brave Idea and Division of Cardiovascular Medicine, Department of Medicine, Brigham and Women’s Hospital, Boston, MA USA; 2 Harvard Medical School, Boston, MA USA; 3 Department of Cardiology, Keio University School of Medicine, Shinjuku, Tokyo Japan; 4 Department of Epidemiology, Harvard T.H. Chan School of Public Health, Boston, MA USA; 5 Division of Cardiology, Feinberg Cardiovascular Research Institute, Northwestern University Feinberg School of Medicine, Chicago, IL USA; 6 Division of Cardiology, Massachusetts General Hospital, Boston, MA USA; 7 Center for Digital Health Innovation and Department of Medicine, University of California San Francisco, San Francisco, CA USA; 8 Department of Preventive Medicine, Northwestern University Feinberg School of Medicine, Chicago, IL USA

**Keywords:** Machine learning, Cardiology

## Abstract

Patients with rare conditions such as cardiac amyloidosis (CA) are difficult to identify, given the similarity of disease manifestations to more prevalent disorders. The deployment of approved therapies for CA has been limited by delayed diagnosis of this disease. Artificial intelligence (AI) could enable detection of rare diseases. Here we present a pipeline for CA detection using AI models with electrocardiograms (ECG) or echocardiograms as inputs. These models, trained and validated on 3 and 5 academic medical centers (AMC) respectively, detect CA with C-statistics of 0.85–0.91 for ECG and 0.89–1.00 for echocardiography. Simulating deployment on 2 AMCs indicated a positive predictive value (PPV) for the ECG model of 3–4% at 52–71% recall. Pre-screening with ECG enhance the echocardiography model performance at 67% recall from PPV of 33% to PPV of 74–77%. In conclusion, we developed an automated strategy to augment CA detection, which should be generalizable to other rare cardiac diseases.

## Abstract (web summary)

Cardiac amyloidosis is difficult to identify, given low prevalence and similarity of the symptoms to more prevalent disorders. Here the authors present a multi-modality, artificial intelligence-enabled pipeline, that enables automated detection of cardiac amyloidosis from inexpensive and accessible measures.

## Introduction

Cardiac amyloidosis arises from deposition of misfolded proteins in the heart muscle, which results in a restrictive-type cardiomyopathy, and commonly progresses to heart failure, conduction system disease, and cardiac death. Cardiac amyloidosis is subclassified based on the specific protein involved, with the major subtypes being transthyretin amyloidosis (ATTR cardiac amyloidosis), caused by misfolding of the transthyretin protein, and light chain amyloidosis (AL cardiac amyloidosis), caused by accumulation of immunoglobulin light chains^1^. Cardiac amyloidosis was previously believed to be rare, but recent reports have suggested that it is largely underdiagnosed^2–6^. The imperative of identifying patients has dramatically increased with the advent of therapies for specific forms of cardiac amyloidosis^7–11^.

The clinical manifestations of cardiac amyloidosis—including conduction system disease, vitreous opacity, carpal tunnel syndrome, orthostatic hypotension, polyneuropathy, spinal stenosis, kidney dysfunction, atrial fibrillation, heart failure—are also commonplace in aging, thus making detection challenging. These signs and symptoms are distributed across multiple organs and tissues (and therefore medical disciplines), and the probabilistic weighting of so many different features is forbidding, even in the unlikely event that all of the relevant exam findings, medical history details and diagnostic test results were available to a given practitioner. Furthermore, definitive diagnostic tests for cardiac amyloidosis—which include tissue biopsy and some forms of radionuclide scintigraphy—are costly and have associated risk, and thus are not plausible as screening approaches^12^.

Cardiac amyloidosis nonetheless has predictive features captured by less expensive and more widely available diagnostic modalities such as electrocardiography^13–16^ (ECG) and echocardiography^17,18^, but the features themselves are not highly specific and thus often missed. Also, some of the recently highlighted echocardiographic features require providers to master specialized software packages^19^, which are time-consuming to use and therefore tend to be employed in practice only after the disease is suspected. A truly generalizable detection strategy should require no specialized acquisition or processing and should rely on only widely available input data. However, the low existing prevalence of the disease places high demands on model performance to reduce the rate of costly false positives, something that has not been achieved to date.

Here, we show a human-interpretation-free machine learning pipeline that accurately detects cardiac amyloidosis using a combination of ECG and echocardiography across multiple institutions.

## Results

### An ECG model detects cardiac amyloidosis effectively across multiple institutions

Electrocardiography is the most widely available cardiac diagnostic test and is frequently performed in primary care settings at a low cost. Since many of the initial manifestations of cardiac amyloidosis are likely to result in a presentation to a primary care physician, we sought first to develop a model based solely on ECG. We constructed ECG-derivation, ECG-validation and ECG-test groups from Brigham and Women’s Hospital (BWH) consisting of 5495, 2247 and 3191 ECG studies respectively (Supplementary Fig. 1, Methods). We tested the model’s performance using data from a held-out partition of the BWH data, as well as distinct cohorts from Massachusetts General Hospital (MGH) and the University of California San Francisco (UCSF), which consisted of 842 and 1,103 studies, respectively (Table 1, Table 2). The composition of AL amyloidosis varied from 34.4% to 58.5% within these groups. There were no patients diagnosed solely based on transthoracic echocardiography (Supplementary Table 1). The dataset included ECGs from various time points before and after a formal diagnosis (Supplementary Fig. 2 and Supplementary Table 2).

**Table 1:** Study-level demographic information (ECG cohort).

	BWH		MGH		UCSF	
	Case	Control	Case	Control	Case	Control
Number of studies	2249	8684	405	437	372	731
Age, years ± SD	69.9 ± 10.4	62.3 ± 13.2	72.9 ± 9.0	73.8 ± 8.8	67.7 ± 12.9	67.5 ± 11.7
Age Groups						
≤30, *n* (%)	2 (0.1)	97 (1.1)	1 (0.2)	1 (0.2)	2 (0.5)	0 (0.0)
30–50, *n* (%)	78 (3.5)	1,370 (15.8)	7 (1.7)	6 (1.4)	36 (9.7)	69 (9,4))
50–70, *n* (%)	901 (40.1)	4548 (52.4)	143 (35.3)	135 (30.9)	136 (36.6)	278 (38.0)
70–90, *n* (%)	1242 (55.2)	2606 (30.0)	254 (62.7)	295 (67.5)	198 (53.2)	384 (52.5)
>90, *n* (%)	26 (1.2)	63 (0.7)	0 (0.0)	0 (0.0)	0 (0.0)	0 (0.0)
HR, bpm ± SD	76.4 ± 16.7	75.9 ± 18.5	78.6 ± 16.6	75.1 ± 19.8	79.6 ± 18.7	72.2 ± 16.3
Sinus rhythm, *n* (%)	1,736 (77.2)	8,072 (93.0)	283 (69.9)	371 (84.9)	365 (98.1)	729 (99.7)

*HR* heart rate, *BWH* Brigham and Women’s Hospital, *MGH* Massachusetts General Hospital, *UCSF* University of California San Francisco. *N* represents the number of studies.

**Table 2:** Patient-level demographic information (ECG cohort).

	BWH		MGH		UCSF	
	Case	Control	Case	Control	Case	Control
Number of patients	480	7,457	52	430	65	725
Age, years ± SD	70.8 ± 9.8	62.2 ± 13.2	71.4 ± 9.4	73.7 ± 8.8	65.5 ± 11.1	67.5 ± 11.7
Age Groups						
≤30, *n* (%)	0 (0.0)	81 (1.1)	0 (0.0)	1 (0.2)	0 (0.0)	0 (0.0)
30–50, *n* (%)	13 (2.7)	1,195 (16.0)	2 (3.8)	6 (1.4)	5 (7.7)	68 (9.4)
50–70, *n* (%)	185 (38.5)	3,929 (52.7)	19 (36.5)	133 (30.9)	34 (52.3)	278 (38.3)
70–90, *n* (%)	277 (57.7)	2,198 (29.5)	31 (59.6)	290 (67.4)	26 (40.0)	379 (52.3)
>90, *n* (%)	5 (1.0)	54 (0.7)	0 (0.0)	0 (0.0)	0 (0.0)	0 (0.0)
Female, *n* (%)	91 (19.0)	2,908 (39.0)	9 (17.3)	48 (11.2)	16 (24.6)	132 (18.2)
Amyloid type						
ATTR, *n* (%)	283 (59.0)	N/A	31 (59.6)	N/A	10 (15.4)	N/A
AL, *n* (%)	165 (34.4)	N/A	15 (38.8)	N/A	38 (58.5)	N/A
Other, *n* (%)	32 (11.5)	N/A	6 (3.8)	N/A	17 (26.2)	N/A

*BWH* Brigham and Women’s Hospital, *MGH* Massachusetts General Hospital, *UCSF* University of California San Francisco. Age on patient level is calculated as mean of all studies for a patient. N/A = not applicable. *N* represents the number of patients.

**Fig. 1:**
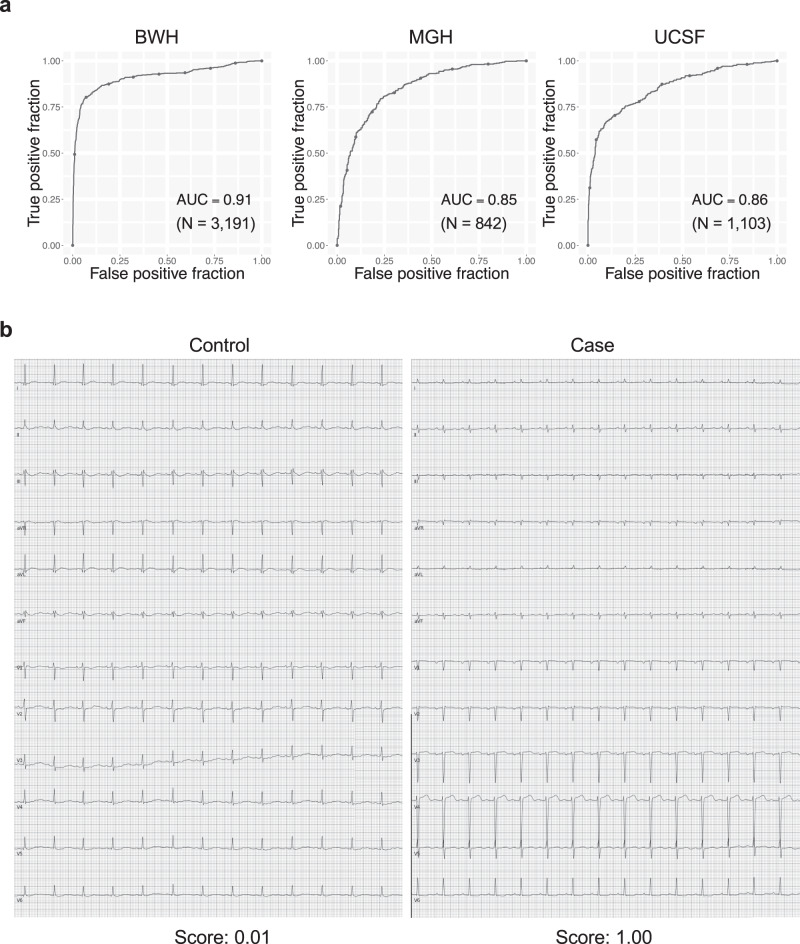
Performance of the cardiac amyloidosis ECG model. **a** ROC plots for detecting cardiac amyloidosis for each institution. The performance on the test dataset is shown for BWH. **b** Representative ECG for cases and controls. The score denotes the model output for the ECG. N is the numbers of studies. Source data are provided as a Source Data file. BWH: Brigham and Women’s Hospital, MGH: Massachusetts General Hospital, UCSF: University of California San Francisco AUC: area under the curve. ECG: electrocardiogram.

The ECG model showed good predictive accuracy as measured by C-statistics of 0.91 (95% CI 0.90–0.93) on the ECG-test set of BWH and similar performance with C-statistics of 0.85 (0.82–0.87) on Massachusetts General Hospital (MGH) cohort and 0.86 (0.83–0.88) for the University of California San Francisco (UCSF) cohort (Fig. 1). The performance was similar when we considered only a single ECG per patient by taking the earliest available ECG, with C-statistics of 0.91 (0.87–0.94), 0.83 (0.78–0.88), and 0.83 (0.77–0.88) on BWH, MGH, and UCSF, respectively (Supplementary Fig. 3). A sensitivity analysis to amyloidosis subtype demonstrated overall similar performance on ATTR amyloid with AUC of 0.92 (0.91–0.94), 0.87 (0.84–0.90), 0.97 (0.95–0.98) when compared to AL amyloid which showed AUC of 0.92 (0.89–0.94), 0.92 (0.89–0.95) and 0.78 (0.75–0.82) for BWH, MGH and the UCSF cohorts, respectively (Supplementary Fig. 4). To determine if our model could detect amyloidosis before a clinical diagnosis was made, we performed a sensitivity analysis limiting cases to time windows before the diagnosis date (e.g., all echocardiograms taken 365 or more days before a diagnosis). This analysis showed that our model was able to detect amyloidosis with C-statistics of 0.88 (0.85–0.92), 0.88 (0.84–0.92), 0.87 (0.82–0.91), 0.87 (0.82–0.91) and 0.88 (0.83–0.92) at 1, 30, 90, 180 and 365 days before the diagnosis date for BWH and 0.88 (0.85–0.91), 0.87 (0.84–0.90), 0.87 (0.84–0.90), 0.87 (0.83–0.90) and 0.85 (0.79–0.89) at 1, 30, 90, 180 and 365 days before the diagnosis date for MGH (Supplementary Fig. 5).

### A video-based echocardiography model for cardiac amyloidosis has very good performance for patients from five AMCs across two countries

Although the ECG-based models were encouraging, we anticipated they did not have the requisite performance characteristics for a low prevalence disease. We thus trained an echocardiography video-based model, using only a single commonly acquired view, the apical 4-chamber view (A4C), which can be collected even with low-cost handheld ultrasound devices. The echocardiography-derivation, echocardiography-validation and echocardiography-test group from BWH had 6,376, 2,684 and 4,117 videos respectively (Supplementary Fig. 6, Methods). The external validation cohorts from MGH, UCSF, Northwestern University (NW), and Keio University Hospital (Keio) in Japan had 441, 369, 229, and 239 studies for 361, 350, 200, and 173 patients, respectively (Tables 3 and 4). As with the ECG cohort, there were no patients diagnosed solely based on transthoracic echocardiography (Supplementary Table 3) and the study dataset included echocardiograms before and after diagnosis **(**Supplementary Fig. 7 and Supplementary Table 4).

**Table 3:** Study-level demographic information (Echocardiogram cohort).

	BWH		MGH		UCSF		NW		Keio	
	Case	Control	Case	Control	Case	Control	Case	Control	Case	Control
Number of studies	1486	3079	110	331	46	323	103	126	118	121
Age, years ± SD	72.9 ± 8.8	65.4 ± 14.5	71.9 ± 9.7	70.2 ± 12.3	70.3 ± 11.6	67.0 ± 12.8	75.1 ± 7.4	74.7 ± 7.6	74.2 ± 8.2	73.8 ± 8.5
Age Groups										
≤30, *n* (%)	0 (0.0)	70 (2.3)	0 (0.0)	0 (0.0)	0 (0.0)	0 (0.0)	0 (0.0)	0 (0.0)	0 (0.0)	0 (0.0)
30–50, *n* (%)	25 (1.7)	381 (12.4)	7 (6.4)	36 (10.9)	3 (6.5)	37 (11.5)	0 (0.0)	0 (0.0)	0 (0.0)	0 (0.0)
50–70, *n* (%)	477 (32.1)	1363 (44.3)	34 (30.9)	108 (32.6)	15 (32.6)	137 (42.4)	27 (26.2)	39 (31.0)	30 (25.4)	34 (28.1)
70–90, *n* (%)	971 (65.3)	1228 (39.9)	69 (62.7)	181 (54.7)	28 (60.9)	149 (46.1)	75 (72.8)	87 (69.0)	86 (72.9)	83 (68.6)
>90, *n* (%)	13 (0.9)	37 (1.2)	0 (0.0)	6 (1.8)	0 (0.0)	0 (0.0)	1 (1.0)	0 (0.0)	2 (1.7)	4 (3.3)
HR, bpm ± SD	74.5 ± 18.4	71.1 ± 18.7	79.4 ± 21.5	71.4 ± 16.1	78.0 ± 14.7	74.1 ± 21.9	74.7 ± 19.5	63.8 ± 9.9	71.8 ± 17.0	69.4 ± 15.4
Manufacture										
Philips, *n* (%)	272 (18.3)	2340 (76.0)	105 (95.5)	313 (94.6)	44 (97.5)	312 (96.6)	0 (0.0)	0 (0.0)	48 (40.7)	38 (31.4)
GE, *n* (%)	1204 (81.0)	724 (23.5)	5 (4.5)	18 (5.4)	2 (4.3)	11 (3.4)	103 (100.0)	126 (100.0)	47 (39.8)	57 (47.1)
Agilent, *n* (%)	3 (0.2)	0 (0.0)	0 (0.0)	0 (0.0)	0 (0.0)	0 (0.0)	0 (0.0)	0 (0.0)	0 (0.0)	0 (0.0)
SonoSite, *n* (%)	1 (0.1)	0 (0.0)	0 (0.0)	0 (0.0)	0 (0.0)	0 (0.0)	0 (0.0)	0 (0.0)	0 (0.0)	0 (0.0)
SIEMENS, *n* (%)	6 (0.4)	15 (0.5)	0 (0.0)	0 (0.0)	0 (0.0)	0 (0.0)	0 (0.0)	0 (0.0)	0 (0.0)	0 (0.0)
TOSHIBA, *n* (%)	0 (0.0)	0 (0.0)	0 (0.0)	0 (0.0)	0 (0.0)	0 (0.0)	0 (0.0)	0 (0.0)	23 (19.5)	26 (21.5)

*HR* heart rate, *BWH* Brigham and Women’s Hospital, *MGH* Massachusetts General Hospital, *UCSF* University of California San Francisco, *NW* Northwestern University, Keio: Keio University Hospital. *N* represents the number of studies.

**Table 4:** Patient-level demographic information (Echocardiogram cohort).

	BWH		MGH		UCSF		NW		Keio	
	Case	Control	Case	Control	Case	Control	Case	Control	Case	Control
Number of patients	410	2418	41	320	32	318	74	126	52	121
Age, years ± SD	72.8 ± 9.4	67.5 ± 13.3	71.0 ± 10.1	70.3 ± 12.4	69.2 ± 12.0	67.0 ± 12.8	75.6 ± 7.3	74.7 ± 7.6	74.2 ± 8.3	73.8 ± 8.5
Age Groups										
≤30, *n* (%)	0 (0.0)	32 (1.3)	0 (0.0)	0 (0.0)	0 (0.0)	0 (0.0)	0 (0.0)	0 (0.0)	0 (0.0)	0 (0.0)
30–50, *n* (%)	8 (2.0)	213 (8.8)	3 (7.3)	36 (11.3)	2 (6.2)	36 (11.3)	0 (0.0)	0 (0.0)	0 (0.0)	0 (0.0)
50–70, *n* (%)	132 (32.2)	1067 (44.1)	14 (34.1)	101 (31.6)	12 (37.5)	136 (42.8)	17 (23.0)	39 (31.0)	13 (25.0)	34 (28.1)
70–90, *n* (%)	265 (64.6)	1074 (44.4)	24 (58.5)	177 (55.3)	18 (56.2)	146 (45.9)	56 (75.7)	87 (69.0)	39 (75.0)	83 (68.6)
>90, *n* (%)	5 (1.2)	37 (1.3)	0 (0.0)	6 (1.9)	0 (0.0)	0 (0.0)	1 (1.4)	0 (0.0)	0 (0.0)	4 (3.3)
Female, *n* (%)	71 (17.3)	843 (34.9)	12 (29.2)	133 (41.6)	10 (31.3)	71 (22.3)	8 (10.8)	14 (11.1)	6 (11.5)	16 (13.2)
Amyloid type										
ATTR, *n* (%)	284 (69.3)	N/A	21 (51.2)	N/A	9 (28.1)	N/A	74 (100.0)	N/A	45 (86.5)	N/A
AL, *n* (%)	124 (30.2)	N/A	17 (41.5)	N/A	11 (34.4)	N/A	0 (0.0)	N/A	7 (13.5)	N/A
Other, *n* (%)	2 (0.5)	N/A	3(2.4)	N/A	12 (37.5)	N/A	0 (0.0)	N/A	0 (0.0)	N/A

*BWH* Brigham and Women’s Hospital, *MGH* Massachusetts General Hospital, *UCSF* University of California San Francisco, *NW* Northwestern University, Keio: Keio University Hospital. *N* represents the number of patients. Age is calculated as mean of all studies for a patient. N represents the number of patients.

**Fig. 2:**
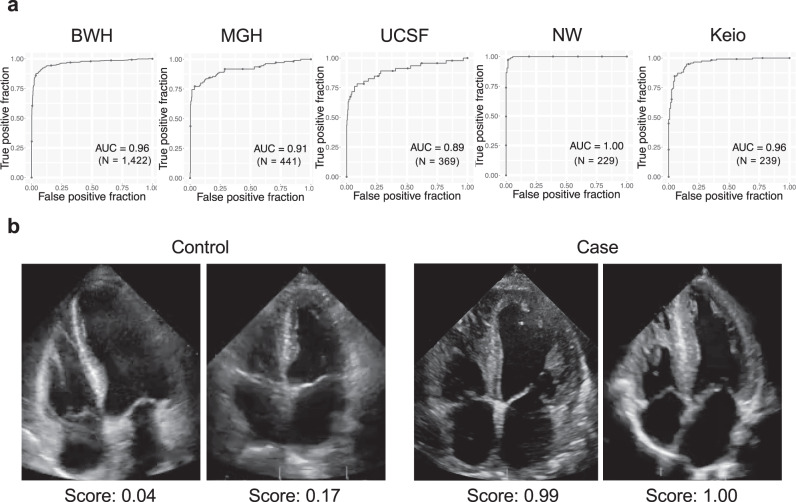
Performance of the cardiac amyloidosis echocardiography model. **a** ROC plots for detecting cardiac amyloidosis for each institution. The performance on the test dataset is shown for BWH. **b** representative echocardiography images for cases and controls. The score denotes the model output for the video. N is the numbers of studies. Source data are provided as a Source Data file. BWH: Brigham and Women’s Hospital, MGH: Massachusetts General Hospital, UCSF: University of California San Francisco, NW: Northwestern University, Keio: Keio University. AUC: area under the curve.

The echocardiography model showed very good predictive accuracy, with C-statistics of 0.96 (0.95–0.97) on the BWH test dataset, and similar performances on external validation cohorts from 3 institutions of US and 1 from Japan with C-statistics of 0.91 (0.88–0.94) for MGH, 0.89 (0.88–0.97) for UCSF, 1.00 (1.00–1.00) for NW and 0.96 (0.91–0.97) for Keio (Fig. 2). This result was similar when taking only the first echocardiogram for each patient with C-statistics of 0.96 (0.94–0.98), 0.93 (0.87–0.98), 0.88 (0.79–0.96), 1.00 (1.00–1.00) and 0.96 (0.92–0.99) on BWH, MGH, UCSF, NW and Keio respectively (Supplementary Fig. 8). Analysis on cardiac amyloidosis subtypes showed superior model performance on ATTR amyloid with C-statistics of 0.97 (0.96–0.98), 0.94 (0.89–0.98), 1.00 (0.99–1.00), 1.00 (1.00–1.00) and 0.96 (0.91–0.98) for BWH, MGH, UCSF, NW, and Keio when compared to AL amyloidosis which had an C-statistics of 0.95 (0.93–0.97), 0.92 (0.87–0.97), 0.84 (0.73–0.93) and 0.95 (0.91–0.98) for BWH, MGH, UCSF and Keio (the NW dataset had no AL amyloidosis cases) (Supplementary Fig. 9). As with the ECG model, we performed a sensitivity analysis by limiting the cases to varying time windows before the diagnosis date. The echocardiography model was also able to detect amyloidosis with very good accuracy with C-statistics of 0.93 (0.90–0.96), 0.91 (0.87–0.95), 0.90 (0.85–0.94), 0.89 (0.84–0.94) and 0.89 (0.83–0.94) at 1, 30, 90, 180 and 365 days before the diagnosis date for BWH, 0.92 (0.85–0.98), 0.91 (0.82–0.98), 0.89 (0.78–0.97), 0.88 (0.76–0.97) and 0.85 (0.70–0.97) at 1, 30, 90, 180 and 365 days before the diagnosis date for MGH and 0.95 (0.91–0.98), 0.94 (0.88–0.98), 0.91 (0.84–0.97), 0.87 (0.79–0.94) and 0.89 (0.82–0.96) at 1, 30, 90, 180 and 365 days before the diagnosis date for Keio (Supplementary Fig. 10).

**Fig. 3:**
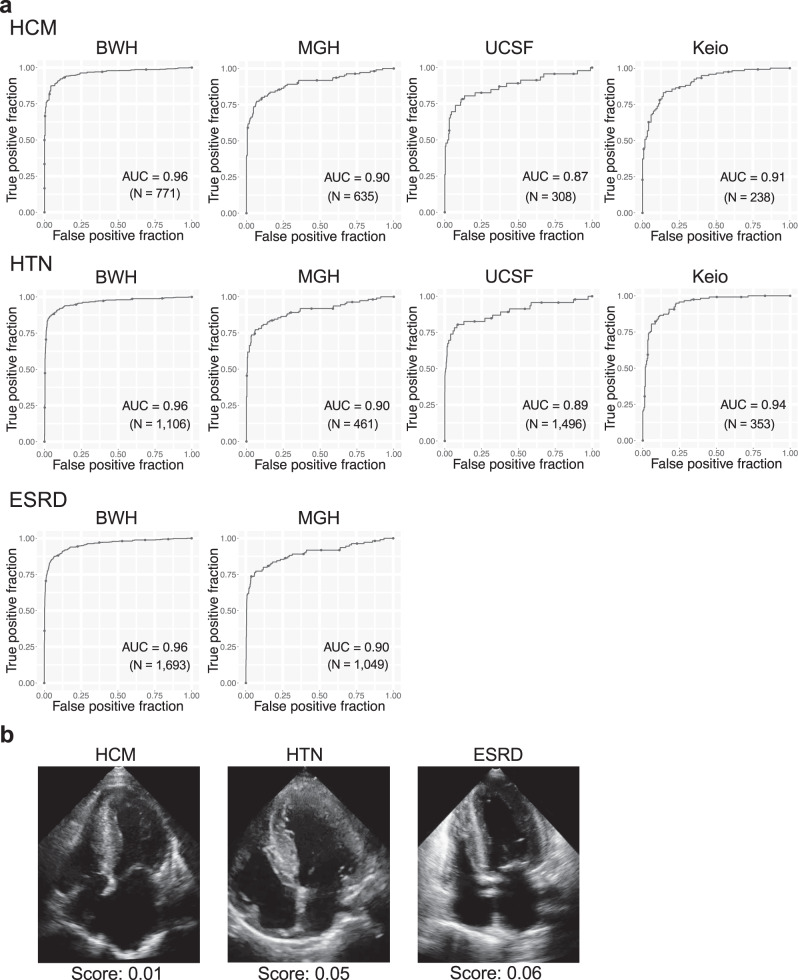
Performance of the echocardiography model for discriminating cardiac amyloidosis from HCM, HTN and ESRD. **a** ROC plots for detecting cardiac amyloidosis for each institution. The performance on the test dataset is shown for BWH-HCM. **b** Representative images for selected controls for each disease. The score denotes the model output for the video. N is the numbers of studies. Source data are provided as a Source Data file. HCM: hypertrophic cardiomyopathy, HTN: hypertension, ESRD: end-stage renal disease. BWH: Brigham and Women’s Hospital, MGH: Massachusetts General Hospital, UCSF: University of California San Francisco, Keio: Keio University. AUC: area under the curve.

To test if our model was able to discriminate cardiac amyloidosis from other diseases that cause cardiac hypertrophy, we further performed analysis by looking at discrimination against patients with hypertrophic cardiomyopathy (HCM), hypertension (HTN) and end-stage renal disease (ESRD) (Supplementary Tables 5 and 6). The model distinguished amyloidosis from these diseases with C-statistics of 0.96 (0.95–0.97), 0.90 (0.86–0.94), 0.87(0.79–0.94) and 0.91 (0.87–0.94) for BWH, MGH, UCSF and Keio dataset respectively for HCM, 0.96 (0.95–0.97), 0.90 (0.86–0.94), 0.89 (0.81–0.95) and 0.94 (0.92–0.96) for BWH, MGH, UCSF and Keio dataset respectively for HTN and 0.96 (0.94–0.97) and 0.90 (0.85–0.93) for BWH and MGH dataset respectively for ESRD (Fig. 3).

### The cardiac amyloidosis echocardiography model outperforms interpretation by expert cardiologists

**Fig. 4:**
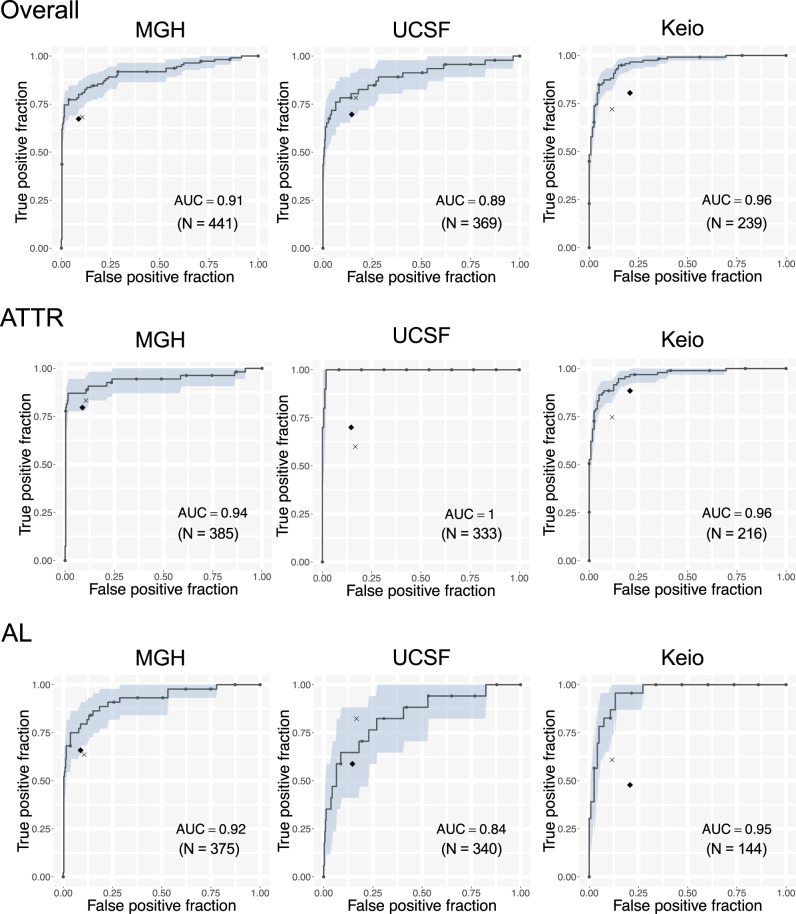
Comparison of the echocardiography model with expert interpretation. ROC plots for detecting cardiac amyloidosis for each institution and amyloid type. The area in light blue represents the 95% CI for the true positive fraction for a given false positive fraction calculated by bootstrap. The black diamond represents the performance of the general cardiologist interpretation and the x represents the performance of the echocardiography expert cardiologist for detecting cardiac amyloidosis. *N* is the numbers of studies. Source data are provided as a Source Data file. ATTR: amyloid transthyretin, AL: amyloid light-chain, MGH: Massachusetts General Hospital, UCSF: University of California San Francisco, Keio: Keio University. AUC: area under the curve.

Two issues make detection of cardiac amyloidosis on echocardiograms particularly challenging for human readers: a lack of sufficiently specific features within the videos and the need to remember to look for these features in every study. Although the latter is difficult to address within existing clinical workflows (though completely solved by an automated system), we sought to evaluate the former by head-to-head comparison. We thus had two expert readers (KM, SG) attempt to diagnose cardiac amyloidosis using the test sets from 3 institutions: MGH, UCSF, and Keio (Fig. 4). In all cases, the model AUC outperformed the human readers (Fig. 4), though for KM on the UCSF data, the result was within the 95% confidence interval. Overall, the model’s superior performance was more apparent for ATTR than AL amyloidosis.

### A stepwise approach using ECG and echocardiography models detects cardiac amyloidosis from a surveillance population

**Fig. 5:**
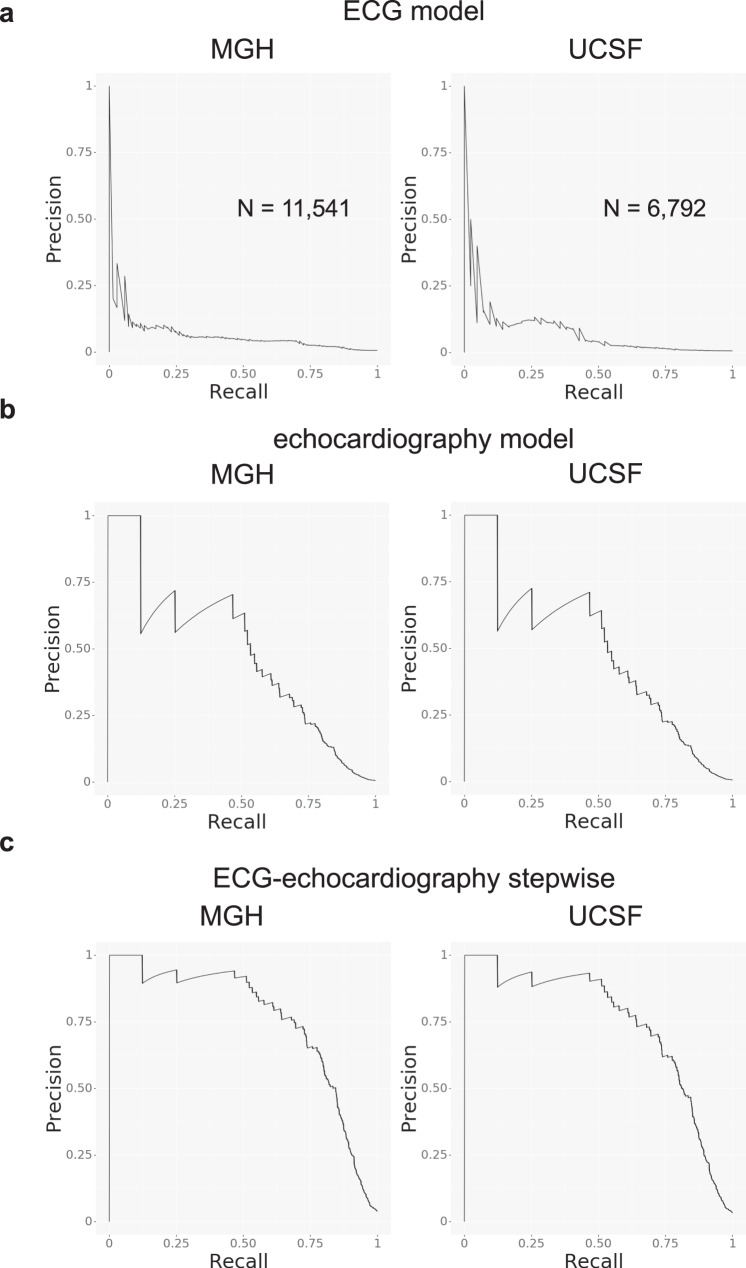
Screening performance of the models on surveillance populations. Precision recall curve plots for **a** the ECG model, **b** the echocardiography model, and **c** the echocardiography model after ECG pre-screening for detection of cardiac amyloidosis in surveillance populations. N is the numbers of studies. Source data are provided as a Source Data file. MGH: Massachusetts General Hospital, UCSF: University of California San Francisco, ECG: electrocardiogram.

Within the MGH and UCSF cohorts, there were 11,541 patients and 6,792 patients with ECG-echocardiogram pairs (within 180 days of one another, with the ECG preceding the echocardiogram), respectively (Table 5). Based on the output of the echocardiography model, we estimated the prevalence of cardiac amyloidosis in this group was 0.60% and 0.62%, which is in keeping with our estimates of cardiac amyloidosis prevalence within this population (see Methods). Using the echocardiography model output as gold standard, the ECG model detected cardiac amyloidosis with PPV 3.9% with recall (i.e,. sensitivity) 71.0% in MGH and PPV 3.4% with recall 52.4% in UCSF at a cutoff of 0.7 (Fig. 5a). Using the ROC curve to estimate a likelihood ratio and the above estimated prevalence numbers, the echocardiography model alone detected cardiac amyloidosis with a PPV of 32.7% with recall 66.9% for MGH and PPV 33.4% with recall 67.0% for UCSF at a cutoff of 0.8 (Fig. 5b). Assuming an updated prevalence after pre-screening using the ECG model, the PPV improved to 76.6% for MGH and 73.9% for UCSF with the same cutoff. The combined ECG-echocardiogram pipeline thus resulted in an overall recall of 47.5 and 34.8% for MGH and UCSF, respectively, at a PPV of nearly 75% (Fig. 5c). In comparison, at a PPV of 75%, the recall values for the echocardiography model alone would be 12.3% for MGH and 12.3% for UCSF.

**Table 5:** Demographic information for deployment simulation cohort.

	MGH	UCSF
Number of patients	11,541	6792
Age, years ± SD	66.0 ± 16.2	61.0 ± 17.8
Age Groups		
≤30, *n* (%)	399 (3.5)	459 (6.8)
30–50, *n* (%)	1419 (12.3)	1328 (19.6)
50–70, *n* (%)	4518 (39.1)	2825 (41.6)
70–90, *n* (%)	4792 (41.5)	2018 (29.7)
>90, *n* (%)	413 (3.6)	162 (2.4)
Female, *n* (%)	5072 (43.9)	3188 (47.0)
HR, bpm ± SD	73.1 ± 18.3	75.2 ± 19.2
Manufacture		
Philips, *n* (%)	11,029 (95.6)	6,165 (90.8)
GE, *n* (%)	512 (4.4)	624 (9.2)
SIEMENS, *n* (%)	0 (0.0)	2 (0.0)
ACUSON, *n* (%)	0 (0.0)	1 (0.0)

*HR* heart rate, *MGH* Massachusetts General Hospital, *UCSF* University of California San Francisco.

## Discussion

Cardiac amyloidosis is one member of a group of cardiovascular diseases, including hypertrophic cardiomyopathy and pulmonary arterial hypertension, that is potentially treatable but rare and therefore difficult to detect^20^. The imperative to recognize patients with these and other rare diseases largely depends on availability of specific therapeutic options, but once these appear, it can be difficult to rapidly adapt prior workflows to ensure that patients are treated appropriately. Moreover, since patients are likely to present to non-experts with their initial symptoms, an operational challenge becomes how best to construct systems that facilitate detection even in such settings^21^.

Although the impact of cardiac amyloidosis on ECG and echocardiography has been known for many decades, the features themselves in isolation have not been sufficiently specific or sensitive to be used as heuristics^15,16,22,23^. For example, in one study of 400 cardiac amyloidosis patients, the characteristic low-voltage ECG pattern of cardiac amyloidosis was seen in only 33% of cardiac amyloidosis patients^13^. One could in principle combine these with other non-cardiac features, but this places an increasing burden on the provider to seek such information, which often only occurs when a suspicion of the disease exists in the first place.

In contrast, the approach we have developed here has deliberately limited the need for any recognition by the provider and use inputs that can be potentially acquired in primary care settings—whether by ECG or handheld echocardiography. To further enable effective deployment in such settings, these detection approaches should ideally be coupled with further facilitation of confirmatory diagnostic processes. In fact, our approach benefits from the fact that there is a second gate of confirmatory diagnostic testing: namely measurement of free light chains, scintigraphy scanning, and possibly tissue biopsy^24^. The ECG and echocardiography models thus represent a tunable detection tool, with cutpoints that can be selected based on population prevalence and costs and benefits (diagnostic, therapeutic, financial and otherwise) of downstream true and false positives (and negatives). The data collected through deployment can itself enable refinement of cutpoints, and potentially spur retraining of models to better match local conditions. Critically, in such a system involving a confirmatory step downstream of the AI detection output, model explainability is less of an issue, and one can focus on maximizing model performance.

There are several limitations to this study. First, since cardiac amyloidosis is an underdiagnosed disease, there may have been undiagnosed cases in the control group. This would produce false labels and may have affected the model performance, as well as the ability to estimate it accurately. For example, false labels in the test sets would worsen the apparent specificity. Second, although our echocardiography model outperformed experts, the expert had access to only the echocardiography videos and no other clinical information. Thus, this analysis compared the ability to detect amyloidosis using only echocardiogram videos but not to a total judgement based on multiple information sources, which are sometimes available in clinical settings.

Medicine has historically reserved screening for widely prevalent diseases such as breast and colon cancer, in part because of the larger number of individuals who may benefit, and also because of the anticipated higher PPV of any diagnostic algorithms. However, given the collective scope of rare diseases^25^, the possibility of developing highly specific models to recognize them (whether by genetics or imaging), and the increasing number of therapies being developed to target them, it will be informative to establish whether a similar paradigm can be developed for other underdiagnosed conditions.

## Methods

### Patient selection procedure for ECG and echocardiography models

For all institutions, prospective cardiac amyloidosis patients were first identified based on diagnostic codes and/or echocardiography reports and then manually confirmed by chart review. Specifically, patients with ATTR cardiac amyloidosis were required to have confirmation of amyloid disease by tissue biopsy, nuclear medicine scan, cardiac magnetic resonance imaging, or genetic testing (transthyretin variant). For AL amyloid, biopsy confirmation was required as well as some evidence of cardiac involvement, whether by cardiac magnetic resonance or echocardiography. The method and date of diagnosis were also identified by chart review. A positive result for myocardial biopsy, cardiac MRI or PYP scan was considered to be diagnostic and the date of whichever study came first defined as the diagnosis date. For cases where providers noted a strong suspicion of amyloidosis on TTE before subsequent confirmation by another modality, the date of the TTE was recorded as the diagnosis date. When notes indicated that the inclusion criteria were met (e.g., statement of “biopsy proven cardiac amyloidosis”) but more details were not available, the method and date of diagnosis was set to “unknown”. For both models, cases were initially matched based on age and sex to patients who underwent ECG or echocardiography at the same institution but did not have cardiac amyloidosis. For the ECG models, we excluded ECGs with pacing spikes.

The ECG model was trained with data from Brigham and Women’s Hospital (BWH) and was externally validated with the data from two different institutions from US: Massachusetts General Hospital (MGH) and University of California San Francisco (UCSF). The patients from BWH were randomly split into three groups (ECG-Derivation, ECG-validation and ECG-Test cohort) in a 5:2:3 ratio to be used for model training (Supplementary Fig. 1). Patients who had ECGs at both BWH and MGH were identified and was allocated to the ECG-Test cohort to avoid overfitting.

The echocardiography model was trained with data from Brigham and Women’s Hospital (BWH) and was externally validated with the data from four different institutions from US and Japan: Massachusetts General Hospital (MGH), University of California San Francisco (UCSF), Northwestern University (NW) and Keio University Hospital (Keio) (Supplementary Figs. 6, 11, 12, 13 and 14). The cases for UCSF were overlapping with those from our previous report^17^. To make the model robust to intracardiac leads and wall thickness, an additional 253 patients with a pacemaker or implantable cardiac defibrillator and without cardiac amyloidosis and 383 patients with HCM were identified and added to the control group for the BWH dataset. The patients from BWH were randomly split into three groups (echocardiography-derivation, echocardiography-validation and echocardiography-test cohort) in a 5:2:3 ratio to be used for model training. Patients who had an echocardiography study at both BWH and MGH were identified and was allocated to the echocardiography-test cohort to avoid overoptimistic estimation of model performance on the MGH test set.

To test the ability of the echocardiography model to discriminate cardiac amyloidosis from other diseases with cardiac hypertrophy, we identified HCM patients in MGH, UCSF, and Keio (Supplementary Figs. 15, 16, 17), HTN patients in BWH, MGH, UCSF, and Keio (Supplementary Figs. 18, 19, 20 and 21), and ESRD patients in BWH and MGH (Supplementary Figs. 22 and 23). HCM patients for BWH, MGH, and Keio were identified by a combination of search by encounter diagnosis and chart review. UCSF HCM patients were taken from those reported previously^17^. HTN for BWH and MGH was defined as a median systolic blood greater than 160 mmHg for blood pressure measurements within two years prior to the echocardiogram study date. For UCSF and Keio, blood pressures were only available within the DICOM header, at the time of the study. ESRD status was defined as patients with an encounter diagnosis ICD-10 code of Z99.2 (dependence on renal dialysis).

### ECG model architecture and training

The ECG model was constructed as a 2D-CNN based model. It consisted of a layer of 2D-CNN followed by 18 layers of multi-2D-CNN-module, which was constructed by 3 parallel multilayer CNNs concatenated at the end of the module (schematic shown in Supplementary Fig. 24, code is included as ECGModel.py). We placed a 50% dropout layer before the final fully connected layer to improve generalization. The model had 49,823,214 parameters total and 49,744,020 were trainable. The model was trained using data from ECG-Derivation cohort from BWH. ECGs were labeled as case=1 or control=0 and the model was trained to minimize the binary cross entropy between model prediction and the label using RMSprop optimizer with initial learning rate of 0.0001. The model was trained for 150 epochs. At the end of each epoch, C-statistics on the ECG-validation cohort were calculated. The final model was chosen as the model with highest C-statistics on the validation cohort across all 150 epochs.

### Echocardiography model architecture and training

Given that echocardiograms are videos, which are time-series of multiple frames, we constructed a 3D-CNN based model treating temporal axis as the 3^rd^ axis rather than taking a frame-by-frame approach as done previously^17^, to maximize the ability of the model to use dynamic features in disease detection. This approach should, in principle, also enable detection of diseases if important features are only visible in a subset of frames. The model consisted of 3 layers of 3D-CNN followed by 12 layers of Multi-3D-CNN-module, which was constructed by 3 parallel multilayer 3D-CNNs and a max pooling operation concatenated at the end of the module (schematic shown in Supplementary Fig. 25, code is included as EchoModel.py). We placed a 40% dropout layer before the final fully connected layer to improve generalization. The scales of the video (in cm/pixel) was input into the fully connected layer. The model had 28,341,385 parameters total and 28,298,105 were trainable. The model was trained using data from echocardiography-derivation cohort from BWH. The echocardiography videos were labeled as case=1 or control=0 at the study level and was trained to minimize the binary cross entropy between model prediction and the label using RMSprop optimizer with initial learning rate of 0.0001. The model was trained for 50 epochs. At the end of each epoch, C-statistics on the echocardiography-validation cohort was calculated. The final model was chosen as the model with highest C-statistics on the validation cohort across all 50 epochs.

### Echocardiography model comparison with expert cardiologist interpretation

The performance of the echocardiography model to detect cardiac amyloidosis was compared with two expert cardiologists (SG: general cardiologist and MK: National Board-certified expert in Adult Comprehensive Echocardiography). The comparison was performed at the study level rather than individual video level. While the CNN model diagnostic output was based on only apical 4 chamber views, the experts had access to all the videos in each echocardiogram study to diagnose cardiac amyloidosis. The experts were blinded to model output. The experts labeled each study as cardiac amyloidosis positive or negative for 3 external validation datasets from MGH, UCSF and Keio. Sensitivity and specificity were calculated and compared with the ROC curve of the model. A subtype analysis on ATTR and AL amyloidosis was also performed.

### Estimating positive predictive value of ECG, echocardiography, and combined ECG-echocardiography models

We estimated prevalence for cardiac amyloidosis within the population of patients with echocardiograms as follows. From our internal data across two large AMCs, we have found that over the past 4 years, 20–25% of the ~16,000–18,000 unique patients who obtain an echocardiogram have at least one encounter diagnosis for heart failure. Of those we anticipate 50% to have heart failure with preserved ejection fraction (HFpEF), or 10–12.5% of patients. The percentage of cardiac amyloidosis within HFpEF is unknown but recent studies suggest proportions of 13–20% in selected subsets^2–6^. Given that these represented enriched populations, we assumed a lower value of 5–7%, which corresponds to 0.5–0.9% of our total population. This value is in keeping with prevalence analysis using 916 successive echocardiograms from Keio University, which included 7 patients with known cardiac amyloidosis (0.76%).

To estimate PPV for our ECG model, we identified 11,541 and 6,792 patients within our respective MGH and UCSF cohorts with an ECG followed by an echocardiogram within 180 days. (Supplementary Figs. 26 and 27). A single ECG-echocardiography study pair was selected for each patient that had the shortest time between ECG and echocardiography studies. We deployed the ECG and echocardiography cardiac amyloidosis models on each study and defined the gold standard as individuals with an echocardiography model score of at least 0.8, a threshold that resulted in prevalence values of 0.60% and 0.62% for MGH and UCSF, respectively. We assessed the ability of ECG model to detect cardiac amyloidosis using precision-recall curve plots.

To assess the PPV for the echocardiography model, we estimated a likelihood ratio from the receiver operating characteristic curve^26^ across the combined test sets for BWH, MGH, UCSF, and Keio. At a threshold of 0.8, the likelihood ratio of the echocardiography model was 83.5. Assuming the above cardiac amyloidosis prevalence of 0.60% and 0.62% for MGH and UCSF, respectively, we were able to estimate an institution-level PPV for the echocardiography model. For the successive deployment of ECG and echocardiography models, we updated the PPV based on the prevalence expected from using only studies that exceeded a cutpoint of 0.7 from the output of the ECG model.

### Statistical analysis

Data were collected and stored using Numpy package version 1.19.2 with Python 3.7.3. All the models were trained with *Keras 2.3.0* on a *Tensorflow 1.14.0* backend^27^. The ROC curves are plotted using the *ggplot2*^28^ package (R 3.6.1) and the C-statistic, sensitivity, specificity, and 95% confidence intervals (using 2000 bootstrap samples) were calculated using the *pROC*^29^ package (1.16.2). The precision-recall plots were made using the *plotnine* package (0.6.0) in Python 3.7.3. Continuous values are presented as mean ± standard deviation (SD) and categorical values are presented as numbers and percentages if not otherwise specified.

### Ethics statement

This study complies with all ethical regulations and guidelines. The study protocol was approved by local institutional review boards (IRB) of Mass General Brigham (2019P002651), UCSF (10–03386), Northwestern University (STU00207540) and Keio University (20200030). This study had minimal patient risk: it collected data retrospectively, there was no direct contact with patients, and data were collected after medical care was completed. Thus, and to recruit an unbiased and representative cohort of patients, data were collected under a waiver of informed consent, which was approved by the IRB. The only minimal risk was breach of confidentiality during data abstraction from the electronic health record system. As such any identifiable health information and study identifier linkage list were securely kept within the original institutions. The model training was done within Mass General Brigham by the authors at that institution (S.G. and R.C.D.). The model validation was run within each institution without sharing identifiable data. All authors had access only to de-identified data during the analysis phase.

### Reporting summary

Further information on research design is available in the Nature Research Reporting Summary linked to this article.

## Supplementary information

Supplementary Information

Reporting Summary

## Data Availability

The data that support the findings of this study are available on request from the corresponding author R.C.D. upon approval of the data sharing committees of the respective institutions. The data are not publicly available due to the presence of information that could compromise research participant privacy. Source data are provided with this paper.

## Source data

Source Data

## Code availability

The code for running the model is attached as a supplementary file. The model weights may contain patient personal information and thus, could not be shared. We provide a web-interface to run our model and generate predictions at http://onebraveideaml.org
