# Supplementary material for: Artificial intelligence-enabled fully automated detection of cardiac amyloidosis using electrocardiograms and echocardiograms
Source: Nat Commun. 2021 May 11;12:2726. doi: 10.1038/s41467-021-22877-8 (PMC8113484; doi:10.1038/s41467-021-22877-8)
Supplement: Supplementary file 1 — Supplementary Information [file 41467_2021_22877_MOESM1_ESM.pdf]

Supplementary Materials

Artificial Intelligence-Enabled Fully Automated Detection of Cardiac Amyloidosis Using Electrocardiograms and Echocardiograms

Shinichi Goto[1,2,5], Keitaro Mahara[3], Lauren Beussink-Nelson[4], Hidehiko Ikura[5], Yoshinori Katsumata[5], Jin Endo[5], Hanna K. Gaggin[2,6], Sanjiv J. Shah[4], Yuji Itabashi[5], Calum A MacRae[1,2], Rahul C Deo[1,2,7,8]*

[1]One Brave Idea and Division of Cardiovascular Medicine, Department of Medicine, Brigham and Women's Hospital, Boston, MA, USA

[2]Harvard Medical School, Boston, MA, USA

[3]Department of Epidemiology, Harvard T.H. Chan School of Public Health, Boston, MA, USA

[4]Division of Cardiology, Feinberg Cardiovascular Research Institute, Northwestern University Feinberg School of Medicine, Chicago, IL, USA

[5]Department of Cardiology, Keio University School of Medicine, Shinjuku, Tokyo, Japan

[6]Division of Cardiology, Massachusetts General Hospital, Boston, MA, USA

[7]Center for Digital Health Innovation and Department of Medicine, University of California San Francisco, San Francisco, CA, USA

[8]Department of Preventive Medicine, Northwestern University Feinberg School of Medicine, Chicago, IL, USA

[*]To whom correspondence should be addressed:

Rahul C. Deo, MD, PhD

One Brave Idea / Division of Cardiovascular Medicine, Department of Medicine, Brigham and Women's Hospital, Boston, MA, USA

rdeo@bwh.harvard.edu

Supplementary Tables

Supplementary Table 1. Method of diagnosis for patients in ECG cohort

	BWH			MGH		
	Overall	ATTR	AL	Overall	ATTR	AL
Number of patients	144	91	47	52	31	15
Method of Diagnosis						
Myocardial biopsy, n (%)	46 (31.9)	13 (27.7)	32 (35.2)	8 (15.4)	6 (19.4)	2 (13.3)
PYP scan, n (%)	44 (30.6)	44 (30.6)	N/A	16 (30.8)	16 (51.6)	N/A
Fat pad biopsy + TTE, n (%)	2 (1.4)	0 (0.0)	2 (4.3)	2 (3.8)	0 (0.0)	2 (13.3)
Cardiac MRI, n (%)	28 (19.4)	10 (11.0)	16 (34.0)	15 (28.8)	7 (22.6)	7 (46.7)
Genetic test, n (%)	1 (0.7)	1 (1.1)	N/A	1 (1.9)	1 (3.2)	N/A
Biopsy (other) + TTE, n (%)	11 (7.6)	0 (0.0)	11 (23.4)	5 (9.6)	0 (0.0)	4 (26.6)
Unknown, n (%)	12 (8.3)	4 (4.3)	5 (10.6)	5 (9.6)	1 (3.2)	0 (0.0)
Days after diagnosis of first ECG						
≤ -365, n (%)	21 (14.6)	15 (16.5)	6 (12.8)	17 (32.7)	14 (45.2)	2 (13.3)
-364 to - 180, n (%)	3 (2.1)	2 (2.2)	1 (2.1)	2 (3.8)	2 (6.5)	0 (0.0)
-179 to -90, n (%)	3 (2.1)	2 (2.2)	1 (2.1)	3 (5.8)	2 (6.5)	0 (0.0)
-89 to -30, n (%)	8 (5.6)	6 (6.6)	2 (4.3)	4 (7.7)	1 (3.2)	3 (20.0)
-29 to -1	18 (12.5)	10 (11.0)	7 (14.9)	4 (7.7)	2 (6.5)	2 (13.3)
0 to 29, n (%)	27 (18.8)	13 (14.3)	14 (29.8)	5 (9.6)	2 (6.5)	3 (20.0)
30 to 89	17 (11.8)	12 (13.2)	3 (6.4)	0 (0.0)	0 (0.0)	0 (0.0)
90 to 179, n (%)	6 (4.2)	6 (6.6)	0 (0.0)	2 (3.8)	0 (0.0)	2 (13.3)
180 to 364, n (%)	9 (6.2)	6 (6.6)	3 (6.4)	2 (3.8)	1 (3.2)	1 (6.7)
365 ≤ , n (%)	17 (11.8)	13 (14.3)	4 (8.5)	7 (13.5)	5 (16.1)	2 (13.3)
Unknown, n (%)	15 (10.4)	6 (6.6)	6 (12.8)	6 (11.5)	0 (0.0)	2 (6.5)

All the patients diagnosed with cardiac MRI was confirmed with either biopsy or PYP scan. The negative value in “Days after diagnosis of first ECG” indicates the date before diagnosis. N represents the number of patients. PYP scan: Tc-99m pyrophosphate scan, TTE: transthoracic echocardiography, ECG: electrocardiogram, MRI: magnetic resonance imaging, ATTR: amyloid transthyretin, AL: amyloid light-chain, BWH: Brigham and Women’s Hospital, MGH: Massachusetts General Hospital.

Supplementary Table 2. Study date relative to diagnosis in ECG cohort

	BWH			MGH		
	Overall	ATTR	AL	Overall	ATTR	AL
Number of studies	669	411	241	405	270	89
Days after diagnosis						
≤ -365, n (%)	98 (14.6)	70 (17.0)	28 (11.6)	54 (13.3)	51 (18.9)	2 (2.2)
-364 to - 180, n (%)	9 (1.3)	8 (1.9)	1 (0.4)	35 (0.6)	35 (13.0)	0 (0.0)
-179 to -90, n (%)	3 (0.4)	2 (0.5)	1 (0.4)	23 (5.7)	21 (7.8)	0 (0.0)
-89 to -30, n (%)	15 (2.2)	11 (2.7)	4 (1.7)	21 (5.2)	10 (3.7)	9 (10.1)
-29 to -1	38 (5.7)	24 (5.8)	10 (4.1)	29 (7.2)	16 (5.9)	12 (13.5)
0 to 29, n (%)	92 (13.8)	35 (8.5)	56 (23.2)	30 (7.4)	20 (7.4)	8 (9.0)
30 to 89	68 (10.2)	27 (6.6)	35 (14.5)	19 (4.7)	14 (5.2)	5 (5.6)
90 to 179, n (%)	34 (5.1)	20 (4.9)	14 (5.8)	20 (4.9)	9 (3.3)	11 (12.4)
180 to 364, n (%)	67 (10.0)	48 (11.7)	18 (7.5)	35 (8.6)	11 (4.1)	23 (25.8)
365 ≤, n (%)	182 (27.2)	133 (32.4)	48 (19.9)	85 (21.0)	65 (24.1)	19 (21.3)
Unknown, n (%)	63 (9.4)	33 (8.0)	26 (10.8)	54 (13.3)	18 (6.7)	0 (0.0)

The negative value in "Days after diagnosis" indicates the date before diagnosis. N represents the number of studies. ECG: electrocardiogram, ATTR: amyloid transthyretin, AL: amyloid light-chain, BWH: Brigham and Women's Hospital, MGH: Massachusetts General Hospital.

Supplementary Table 3. Method of diagnosis for patients in echocardiogram cohort

	BWH			MGH			Keio		
	Overall	ATTR	AL	Overall	ATTR	AL	Overall	ATTR	AL
Number of patients	134	96	38	41	21	17	52	45	7
Method of Diagnosis									
Myocardial biopsy, n (%)	34 (25.4)	23 (24.0)	11 (28.9)	6 (14.6)	3 (14.3)	3 (17.6)	36 (69.2)	34 (75.6)	2 (28.6)
PYP scan, n (%)	55 (41.0)	55 (57.3)	N/A	11 (26.8)	11 (52.4)	N/A	11 (21.2)	11 (24.4))	N/A
Fat pad biopsy + TTE, n (%)	0 (0.0)	0 (0.0)	0 (0.0)	2 (4.9)	0 (0.0)	2 (11.8)	3 (5.8)	0 (0.0)	3 (42.9)
Cardiac MRI, n (%)	35 (26.1)	13 (13.5)	35 (26.1)	15 (36.6)	7 (33.3)	7 (41.2)	0 (0.0)	0 (0.0)	0 (0.0)
Genetic test, n (%)	1 (0.7)	1 (1.0)	N/A	0 (0.0)	0 (0.0)	N/A	0 (0.0)	0 (0.0)	0 (0.0)
Biopsy (other) + TTE, n (%)	6 (4.5)	2 (2.1)	3 (7.9)	5 (12.2)	0 (0.0)	5 (29.4)	2 (3.8)	0 (0.0)	2 (28.6)
Unknown, n (%)	3 (2.2)	2 (2.1)	1 (0.7)	2 (4.9)	0 (0.0)	0 (0.0)	0 (0.0)	0 (0.0)	0 (0.0)
Days after diagnosis of first echocardiogram									
≤ -365, n (%)	22 (16.4)	11 (11.5)	11 (28.9)	9 (22.0)	8 (38.1)	0 (0.0)	2 (3.8)	2 (4.4)	0 (0.0)
-364 to - 180, n (%)	5 (3.7)	4 (4.2)	1 (2.6)	2 (4.9)	2 (9.5)	0 (0.0)	2 (3.8)	0 (0.0)	2 (28.6)
-179 to -90, n (%)	2 (1.5)	0 (0.0)	2 (2.1)	0 (0.0)	0 (0.0)	0 (0.0)	3 (5.8)	2 (4.4)	1 (14.3)
-89 to -30, n (%)	3 (2.2)	0 (0.0)	3 (3.1)	4 (9.8)	1 (4.8)	3 (17.6)	10 (19.2)	8 (17.8)	2 (28.6)
-29 to -1	14 (10.4)	2 (5.3)	12 (12.5)	6 (14.6)	2 (9.5)	4 (23.5)	10 (19.2)	8 (17.8)	2 (28.6)
0 to 29, n (%)	24 (17.9)	15 (15.6)	9 (23.7)	1 (2.4)	0 (0.0)	1 (5.9)	0 (0.0)	0 (0.0)	0 (0.0)
30 to 89	12 (9.0)	11 (11.5)	1 (2.6)	0 (0.0)	0 (0.0)	0 (0.0)	4 (7.7)	4 (8.9)	0 (0.0)
90 to 179, n (%)	10 (7.5)	8 (8.3)	2 (5.3)	4 (9.8)	0 (0.0)	4 (23.5)	4 (7.7)	4 (8.9)	0 (0.0)
180 to 364, n (%)	14 (10.4)	9 (9.4)	5 (13.2)	4 (9.8)	1 (4.8)	3 (17.6)	11 (21.2)	11 (24.4)	0 (0.0)
365 ≤, n (%)	24 (17.9)	18 (18.8)	6 (15.8)	9 (22.0)	1 (4.8)	2 (11.8)	6 (11.5)	6 (13.3)	0 (0.0)
Unknown, n (%)	4 (3.0)	3 (3.1)	1 (2.6)	2 (4.9)	0 (0.0)	0 (0.0)	0 (0.0)	0 (0.0)	0 (0.0)

All the patients diagnosed with cardiac MRI was confirmed with either biopsy or PYP scan. The negative value in “Days after diagnosis of first echocardiogram” indicates the date before diagnosis. N represents the number of patients. PYP scan: Tc-99m pyrophosphate scan, TTE: transthoracic echocardiography, MRI: magnetic resonance imaging, ATTR: amyloid transthyretin, AL: amyloid light-chain, BWH: Brigham and Women’s Hospital, MGH: Massachusetts General Hospital, Keio: Keio University.

Supplementary Table 4. Study date relative to diagnosis in echocardiogram cohort

	BWH			MGH			Keio		
	Overall	ATTR	AL	Overall	ATTR	AL	Overall	ATTR	AL
Number of studies	513	362	151	110	54	44	118	95	23
Days after diagnosis									
≤ -365, n (%)	39 (7.6)	16 (4.4)	23 (15.2)	18 (16.4)	16 (29.6)	0 (0.0)	4 (3.4)	4 (4.2)	0 (0.0)
-364 to - 180, n (%)	15 (2.9)	5 (1.4)	10 (6.6)	7 (6.4)	7 (13.0)	0 (0.0)	2 (1.7)	0 (0.0)	2 (8.7)
-179 to -90, n (%)	7 (1.4)	5 (1.4)	2 (1.3)	2 (1.8)	1 (1.9)	0 (0.0)	3 (2.5)	2 (2.1)	1 (4.3)
-89 to -30, n (%)	6 (1.2)	5 (1.4)	1 (0.7)	6 (5.5)	3 (5.6)	3 (6.8)	11 (9.3)	8 (8.4)	3 (13.0)
-29 to -1	24 (4.7)	19 (5.2)	5 (3.3)	8 (7.3)	4 (7.4)	4 (9.1)	13 (11.0)	9 (9.5)	4 (17.4)
0 to 29, n (%)	35 (6.8)	19 (5.2)	16 (10.6)	5 (4.5)	2 (3.7)	2 (4.5)	6 (5.1)	5 (5.3)	1 (4.3)
30 to 89	23 (4.5)	18 (5.0)	5 (3.3)	4 (3.6)	0 (0.0)	4 (9.1)	11 (9.3)	6 (6.3)	5 (21.7)
90 to 179, n (%)	23 (4.5)	15 (4.1)	8 (5.3)	7 (6.4)	1 (1.9)	6 (13.6)	8 (6.8)	8 (8.4)	0 (0.0)
180 to 364, n (%)	58 (11.3)	44 (12.2)	14 (9.3)	11 (10.0)	4 (7.4)	7 (15.9)	21 (17.8)	19 (20.0)	2 (8.7)
365 ≤, n (%)	267 (52.0)	204 (56.4)	63 (41.7)	34 (30.9)	16 (29.6)	18 (40.9)	39 (33.1)	34 (35.8)	5 (21.7)
Unknown, n (%)	16 (3.1)	12 (3.3)	4 (2.6)	8 (7.3)	0 (0.0)	0 (0.0)	0 (0.0)	0 (0.0)	0 (0.0)

The negative value in "Days after diagnosis" indicates the date before diagnosis. N represents the number of studies. ATTR: amyloid transthyretin, AL: amyloid light-chain, BWH: Brigham and Women's Hospital, MGH: Massachusetts General Hospital, Keio: Keio University.

Supplementary Table 5. Study-level demographic information of controls of HCM, HTN and ESRD

	BWH			MGH			UCSF		Keio	
	HCM	ESRD	HTN	HCM	ESRD	HTN	HCM	HTN	HCM	HTN
Number of studies	258	1,180	593	525	939	351	262	1,450	120	235
Age, years ± SD	55.1 ± 13.9	60.1 ± 15.2	65.3 ± 18.4	60.1 ± 15.2	63.6 ± 14.7	70.1 ± 15.1	58.9 ± 14.9	66.7 ± 16.7	59.6 ± 13.5	73.1 ± 13.1
Age Groups										
≤30, n (%)	13 (5.0)	45 (3.8)	43 (7.3)	24 (4.6)	17 (1.8)	6 (1.7)	11 (4.2)	69 (4.8)	3 (2.5)	2 (0.9)
30-50, n (%)	72 (27.9)	234 (19.8)	55 (9.3)	93 (17.7)	157 (16.7)	21 (6.0)	64 (24.4)	138 (9.5)	23 (19.2)	14 (6.0)
50-70, n (%)	137 (53.1)	532 (45.1)	236 (39.8)	264 (50.3)	396 (42.2)	136 (38.7)	126 (48.1)	593 (40.9)	64 (53.3)	67 (28.5)
70-90, n (%)	36 (14.0)	369 (31.3)	236 (39.8)	141 (26.9)	353 (37.6)	165 (47.0)	60 (22.9)	594 (41.0)	30 (25.0)	139 (59.1)
>90, n (%)	0 (0.0)	0 (0.0)	23 (3.9)	3 (0.6)	16 (1.7)	23 (6.6)	1 (0.4)	56 (3.9)	0 (0.0)	13 (5.5)
HR, bpm ± SD	70.6 ± 16.6	77.8 ± 18.7	68.9 ± 14.5	65.8 ± 15.6	75.5 ± 17.9	67.8 ± 16.1	64.8 ± 17.3	72.1 ± 17.7	61.5 ± 13.1	67.3 ± 12.7
Manufacture										
Philips, n (%)	201 (77.9)	867 (73.5)	432 (72.8)	487 (92.8)	910 (96.9)	334 (95.2)	182 (69.5)	1,158 (79.9)	4 (3.3)	64 (27.2)
GE, n (%)	55 (21.3)	296 (25.1)	160 (27.0)	36 (6.9)	29 (3.1)	17 (4.8)	80 (30.5)	290 (20.0)	115 (95.8)	131 (55.7)
Agilent, n (%)	1 (0.1)	1 (0.1)	0 (0.0)	0 (0.0)	0 (0.0)	0 (0.0)	0 (0.0)	0 (0.0)	0 (0.0)	0 (0.0)
SonoSite, n (%)	1 (0.1)	1 (0.1)	0 (0.0)	0 (0.0)	0 (0.0)	0 (0.0)	0 (0.0)	0 (0.0)	0 (0.0)	0 (0.0)
SIEMENS, n (%)	3 (0.4)	7 (0.6)	1 (0.2)	0 (0.0)	0 (0.0)	0 (0.0)	0 (0.0)	1 (0.1)	0 (0.0)	0 (0.0)
ACUSON, n (%)	0 (0.0)	0 (0.0)	0 (0.0)	0 (0.0)	0 (0.0)	0 (0.0)	0 (0.0)	1 (0.1)	0 (0.0)	0 (0.0)
ATL, n (%)	0 (0.0)	0 (0.0)	0 (0.0)	2 (0.4)	0 (0.0)	0 (0.0)	0 (0.0)	0 (0.0)	0 (0.0)	0 (0.0)
TOSHIBA, n (%)	0 (0.0)	0 (0.0)	0 (0.0)	0 (0.0)	0 (0.0)	0 (0.0)	0 (0.0)	0 (0.0)	1 (0.8)	40 (17.0)

N represents the number of studies. HR: heart rate, BWH: Brigham and Women's Hospital, MGH: Massachusetts General Hospital, UCSF: University of California San Francisco, Keio: Keio University. HCM: hypertrophic cardio myopathy. ESRD: end-stage renal disease. HTN: hypertension.

Supplementary Table 6. Patient-level demographic information of controls of HCM, HTN and ESRD

	BWH			MGH			UCSF		Keio	
	HCM	ESRD	HTN	HCM	ESRD	HTN	HCM	HTN	HCM	HTN
Number of patients	114	528	538	243	476	318	159	1,360	54	228
Age, years ± SD	55.8 ± 13.6	60.9 ± 15.1	65.0 ± 18.2	59.6 ± 15.1	64.0 ± 14.3	69.6 ± 15.2	57.3 ± 15.8	66.7 ± 16.7	60.7 ± 13.3	72.9 ± 13.2
Age Groups										
≤30, n (%)	6 (5.3)	21 (4.0)	40 (7.4)	13 (5.3)	10 (2.1)	6 (1.9)	11 (6.9)	68 (5.0)	1 (1.9)	2 (0.9)
30-50, n (%)	30 (26.3)	99 (18.8)	50 (9.3)	44 (18.1)	67 (14.1)	20 (6.3)	41 (25.8)	129 (9.5)	10 (18.5)	14 (6.1)
50-70, n (%)	65 (57.0)	234 (44.3)	219 (40.7)	122 (50.2)	214 (45.0)	124 (39.0)	74 (46.5)	554 (40.7)	28 (51.9)	66 (28.9)
70-90, n (%)	13 (11.4)	174 (33.0)	208 (38.7)	63 (25.9)	179 (37.6)	150 (47.2)	33 (20.8)	554 (40.7)	15 (27.8)	134 (58.8)
>90, n (%)	0 (0.0)	0 (0.0)	21 (3.9)	1 (0.4)	6 (1.3)	18 (5.7)	0 (0.0)	55 (4.0)	0 (0.0)	12 (5.3)
Female, n (%)	35 (30.7)	222 (42.0)	253 (47.0)	84 (34.6)	162 (34.0)	155 (48.7)	68 (42.8)	683 (50.4)	21 (38.9)	119 (52.2)

N represents the number of patients. BWH: Brigham and Women's Hospital, MGH: Massachusetts General Hospital, UCSF: University of California San Francisco, Keio: Keio University Hospital. Age is calculated as mean of all studies for a patient. HCM: hypertrophic cardio myopathy. ESRD: end-stage renal disease. HTN: hypertension.

Supplementary Figures.

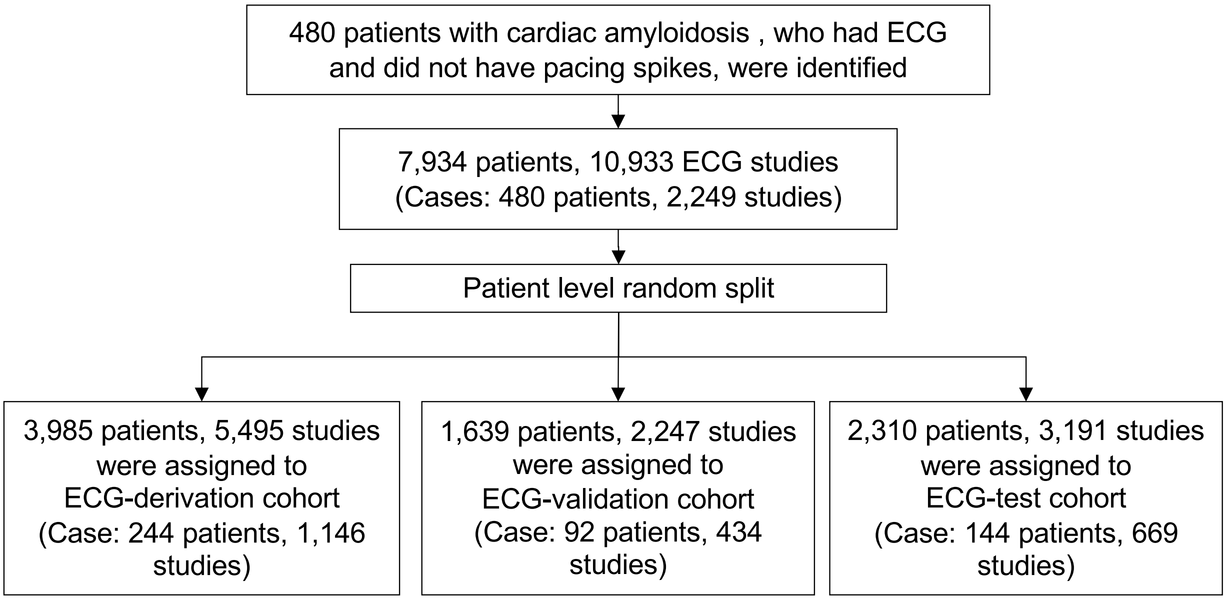

Supplementary Figure 1. Cohort selection procedure for the BWH ECG cohort.

ECG: electrocardiogram, BWH: Brigham and Women's Hospital.

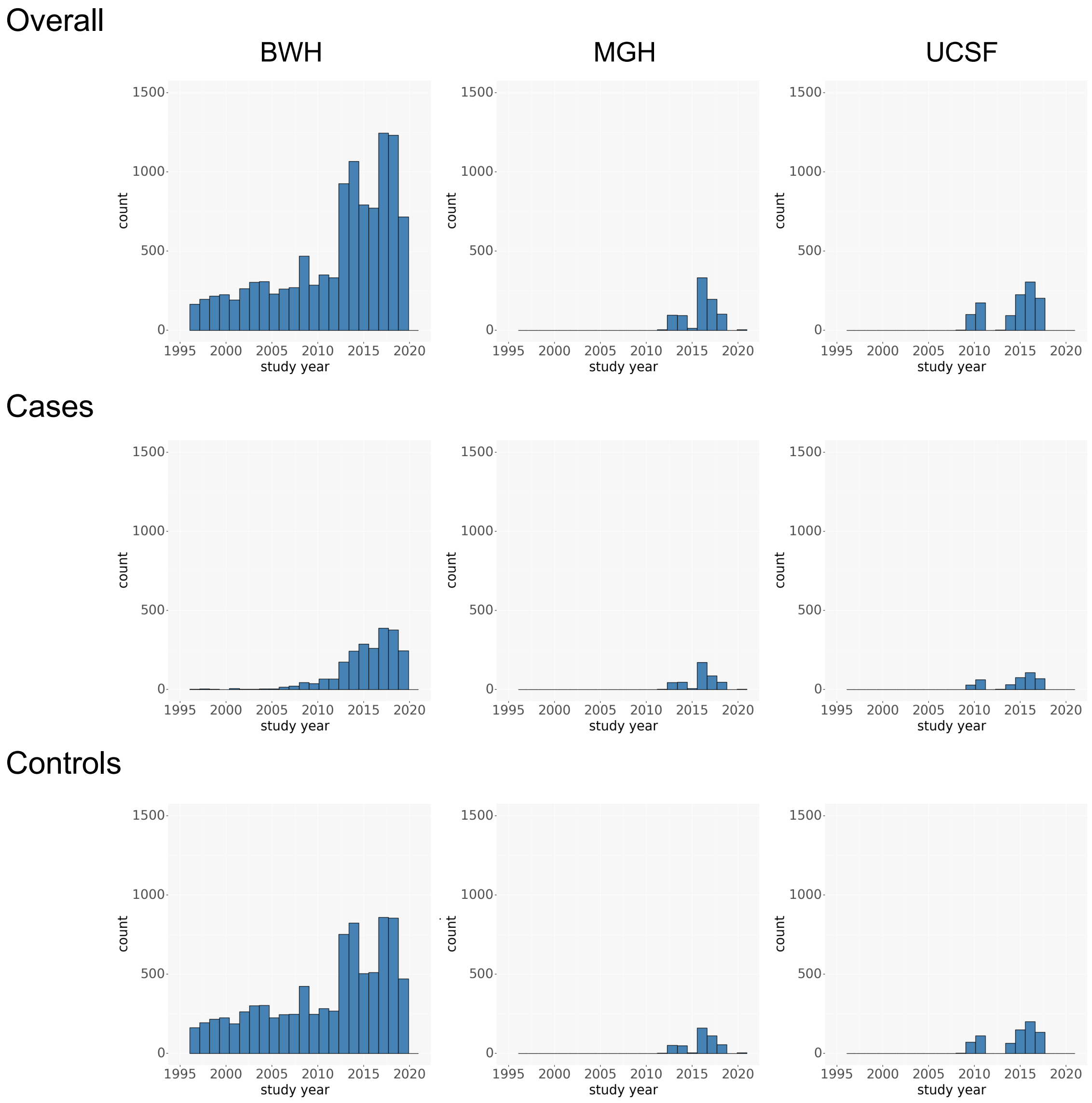

Supplementary Figure 2. Histogram of the dates of ECG studies

Source data are provided as a Source Data file. ECG: electrocardiogram, BWH: Brigham and Women’s Hospital, MGH: Massachusetts General Hospital, UCSF: University of California San Francisco.

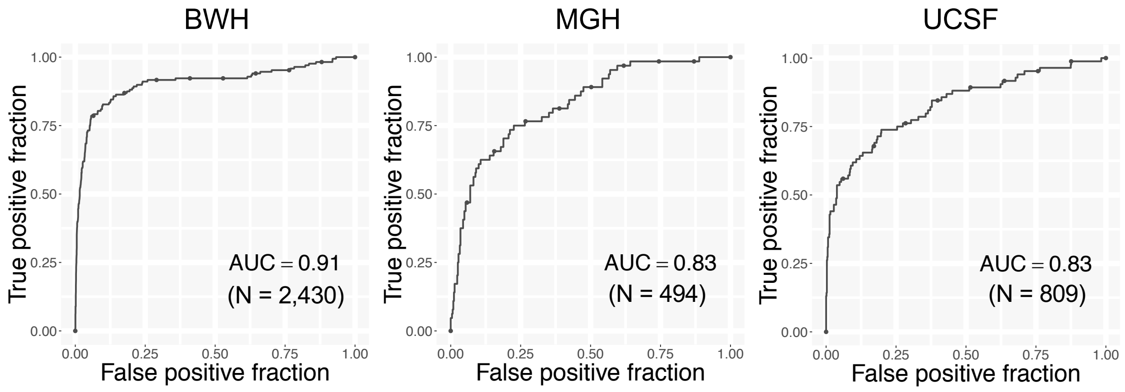

Supplementary Figure 3. Performance of the ECG model on the earliest available study for each patient

ROC plots for detecting cardiac amyloidosis for each institution. The performance on the test dataset is shown for BWH. Source data are provided as a Source Data file. ECG: electrocardiogram, BWH: Brigham and Women's Hospital, MGH: Massachusetts General Hospital, UCSF: University of California San Francisco. AUC: area under the curve.

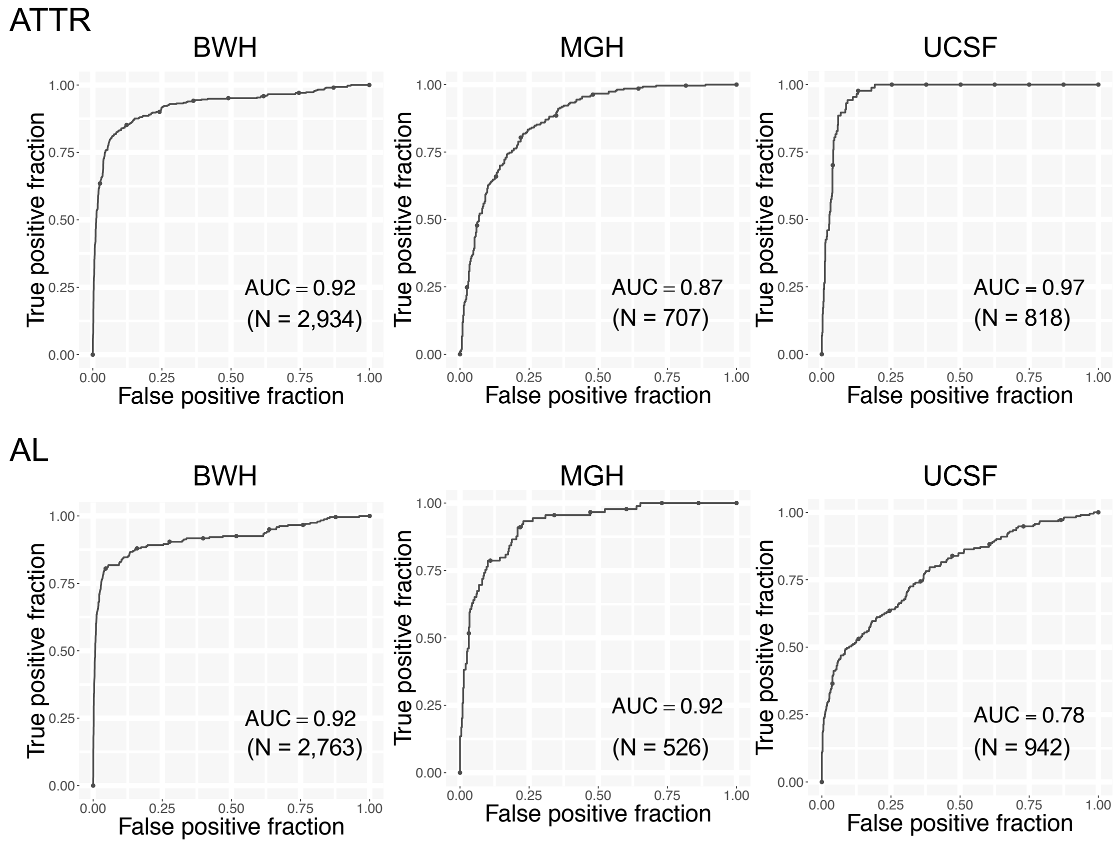

Supplementary Figure 4. Performance of ECG model by amyloid subtype

ROC plots for detecting cardiac amyloidosis on amyloid subtype for each institution. The performance on a test dataset is shown for BWH. N is the numbers of studies. Source data are provided as a Source Data file. ECG: electrocardiogram, ATTR: amyloid transthyretin, AL: amyloid light-chain, BWH: Brigham and Women's Hospital, MGH: Massachusetts General Hospital, UCSF: University of California San Francisco. AUC: area under the curve.

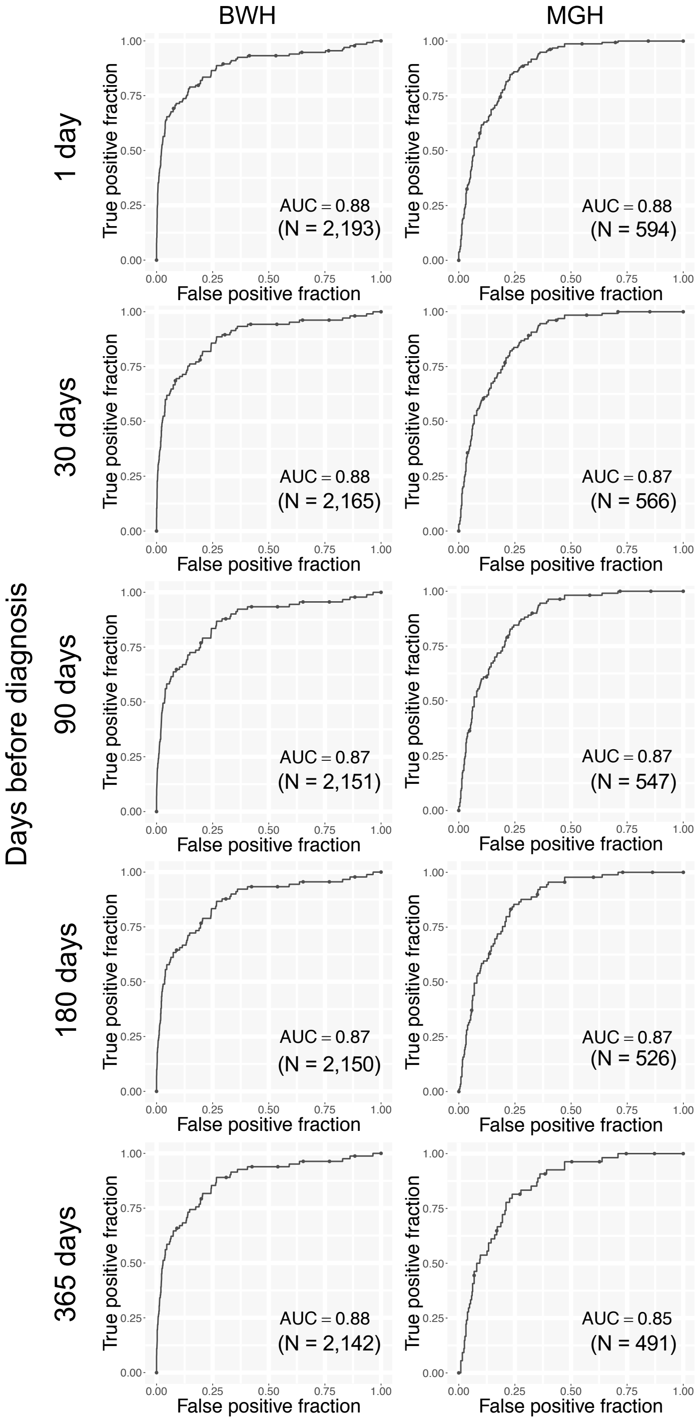

Supplementary Figure 5. Performance of the ECG model using studies collected up to various time points prior to the diagnosis.

ROC plots for detecting cardiac amyloidosis on datasets for each institution. The performance on a test dataset is shown for BWH. Source data are provided as a Source Data file. ECG: electrocardiogram, BWH: Brigham and Women's Hospital, MGH: Massachusetts General Hospital, AUC: area under the curve.

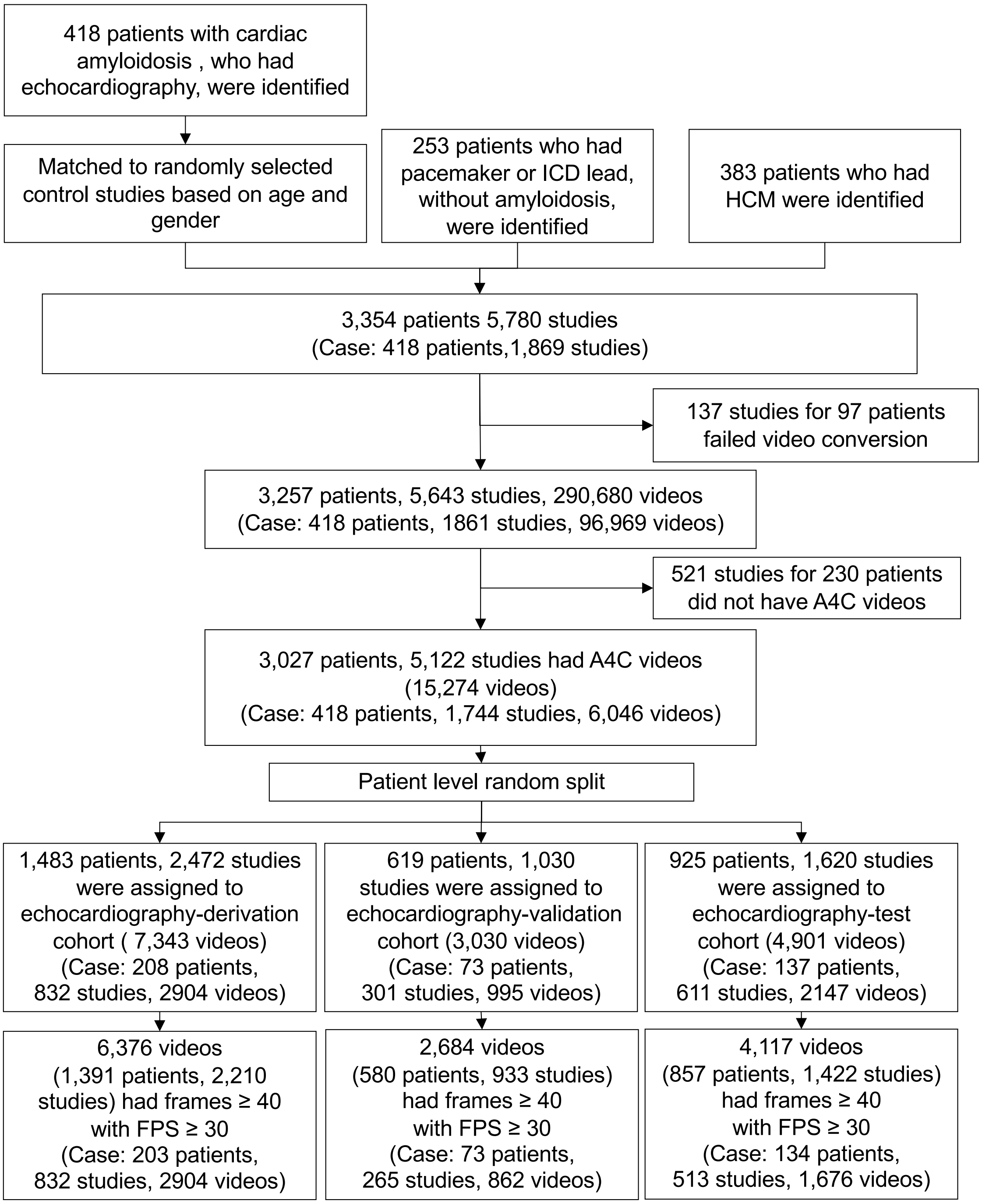

Supplementary Figure 6. Cohort selection procedure for the BWH echocardiography cohort.

BWH: Brigham and Women's Hospital, HCM: hypertrophic cardiomyopathy, ICD: implantable cardioverter defibrillator, FPS: frames per second, A4C: apical 4 chamber view.

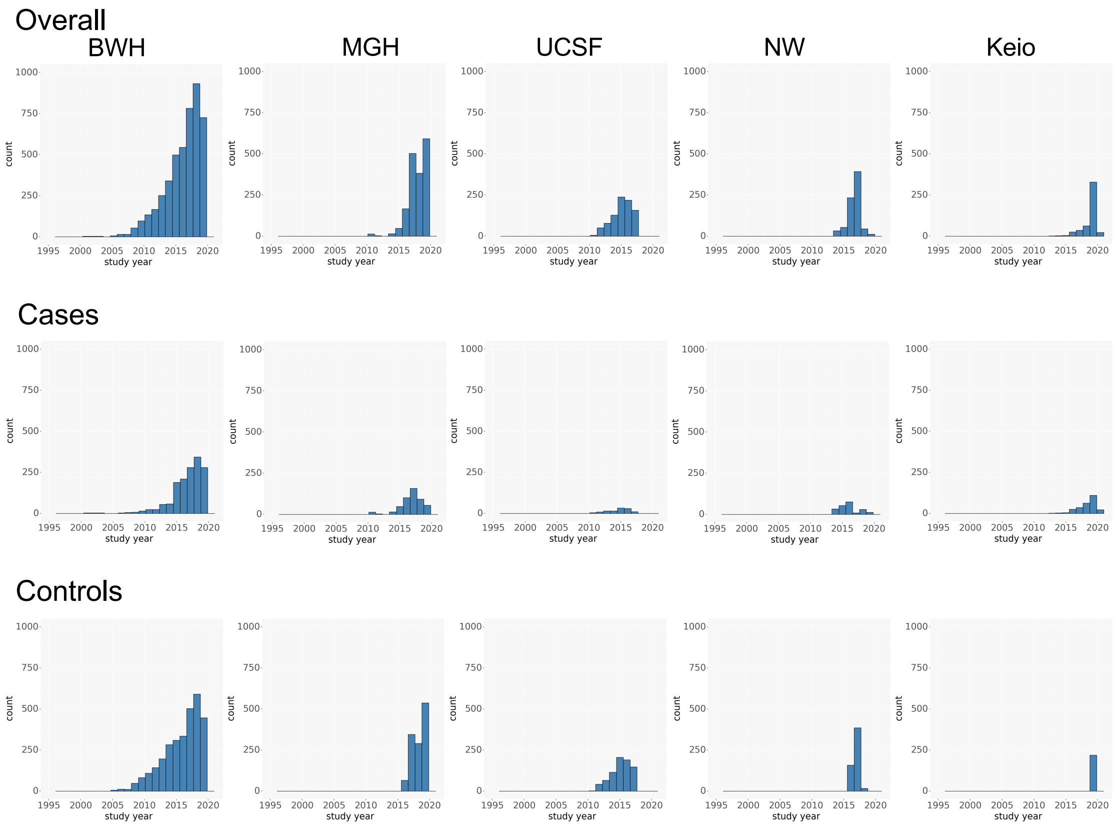

Supplementary Figure 7. Histogram of the dates of echocardiology studies

Source data are provided as a Source Data file. BWH: Brigham and Women's Hospital, MGH: Massachusetts General Hospital, UCSF: University of California San Francisco, NW: Northwestern University, Keio: Keio University.

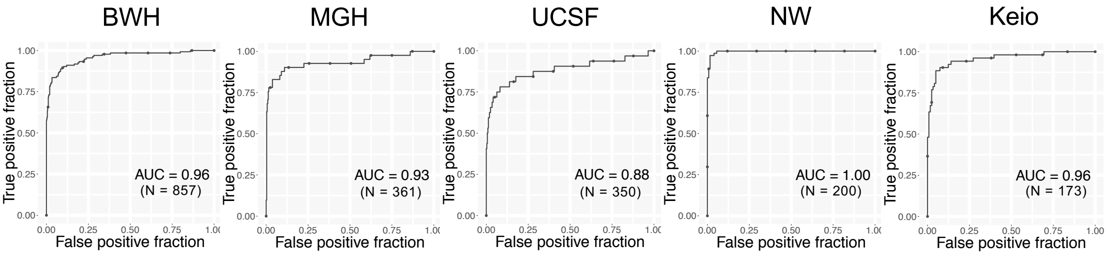

Supplementary Figure 8. Performance of the echocardiography model using the earliest available study for each patient

ROC plots for detecting cardiac amyloidosis for each institution. The performance on the test dataset is shown for BWH. N is the numbers of studies. Source data are provided as a Source Data file. BWH: Brigham and Women's Hospital, MGH: Massachusetts General Hospital, UCSF: University of California San Francisco, NW: Northwestern University, Keio: Keio University. AUC: area under the curve.

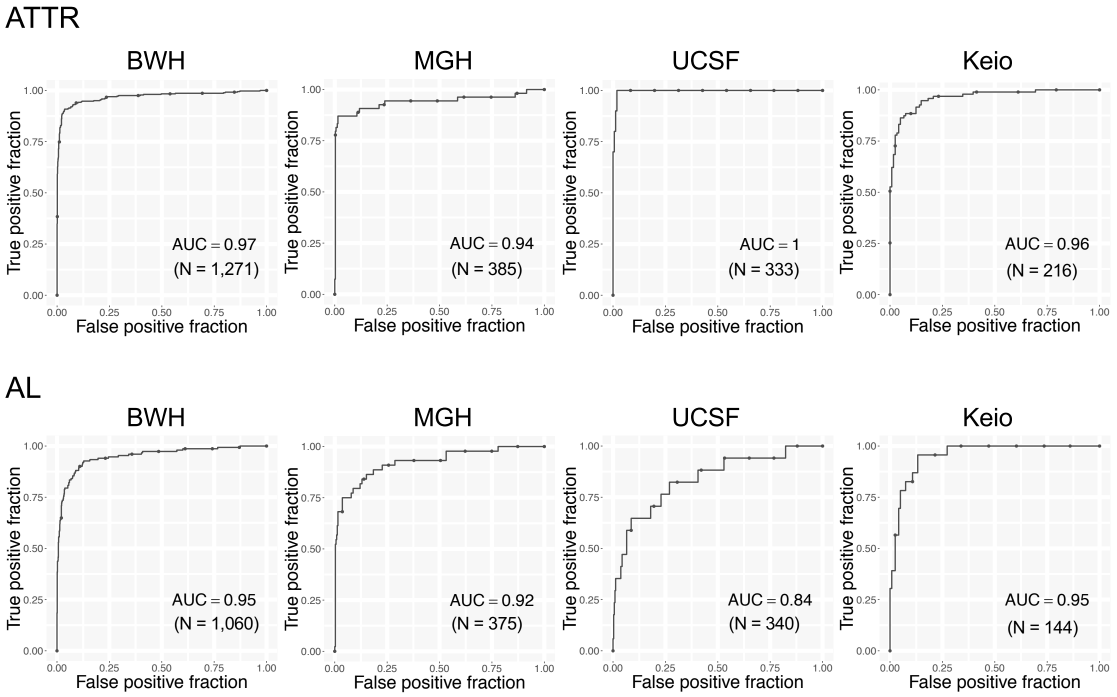

Supplementary Figure 9. Performance of echocardiography model by amyloid subtype

ROC plots for detecting cardiac amyloidosis by subtype for each institution. The performance on a test dataset is shown for BWH. N is the numbers of studies. Source data are provided as a Source Data file. ATTR: amyloid transthyretin, AL: amyloid light-chain, BWH: Brigham and Women's Hospital, MGH: Massachusetts General Hospital, UCSF: University of California San Francisco, Keio: Keio University, AUC: area under the curve.

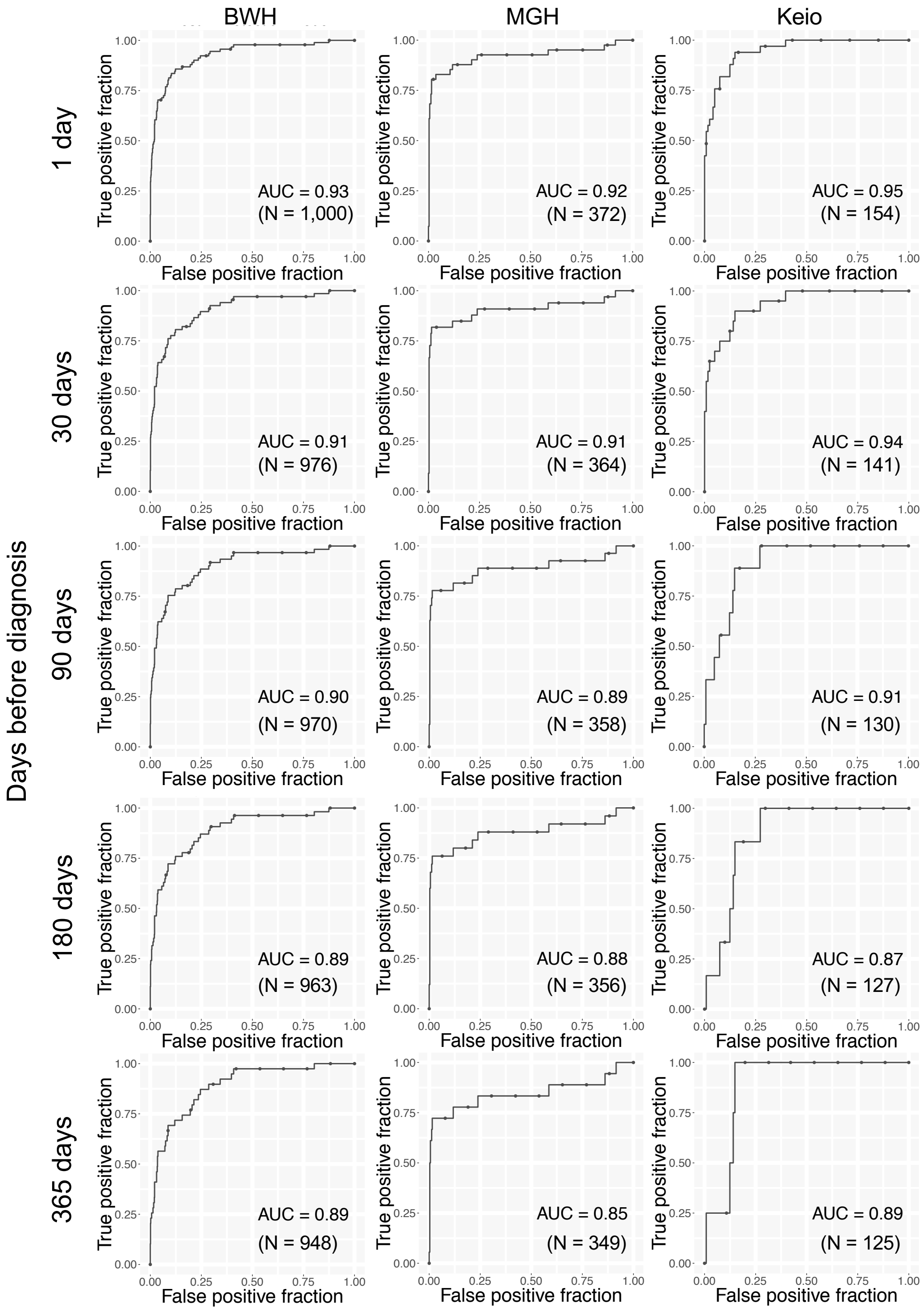

Supplementary Figure 10. Performance of the echocardiography model on the studies from various time points before diagnosis

ROC plots for detecting cardiac amyloidosis for each institution. The performance on the test dataset is shown for BWH. Source data are provided as a Source Data file. BWH: Brigham and Women's Hospital, MGH: Massachusetts General Hospital, Keio: Keio University. AUC: area under the curve.

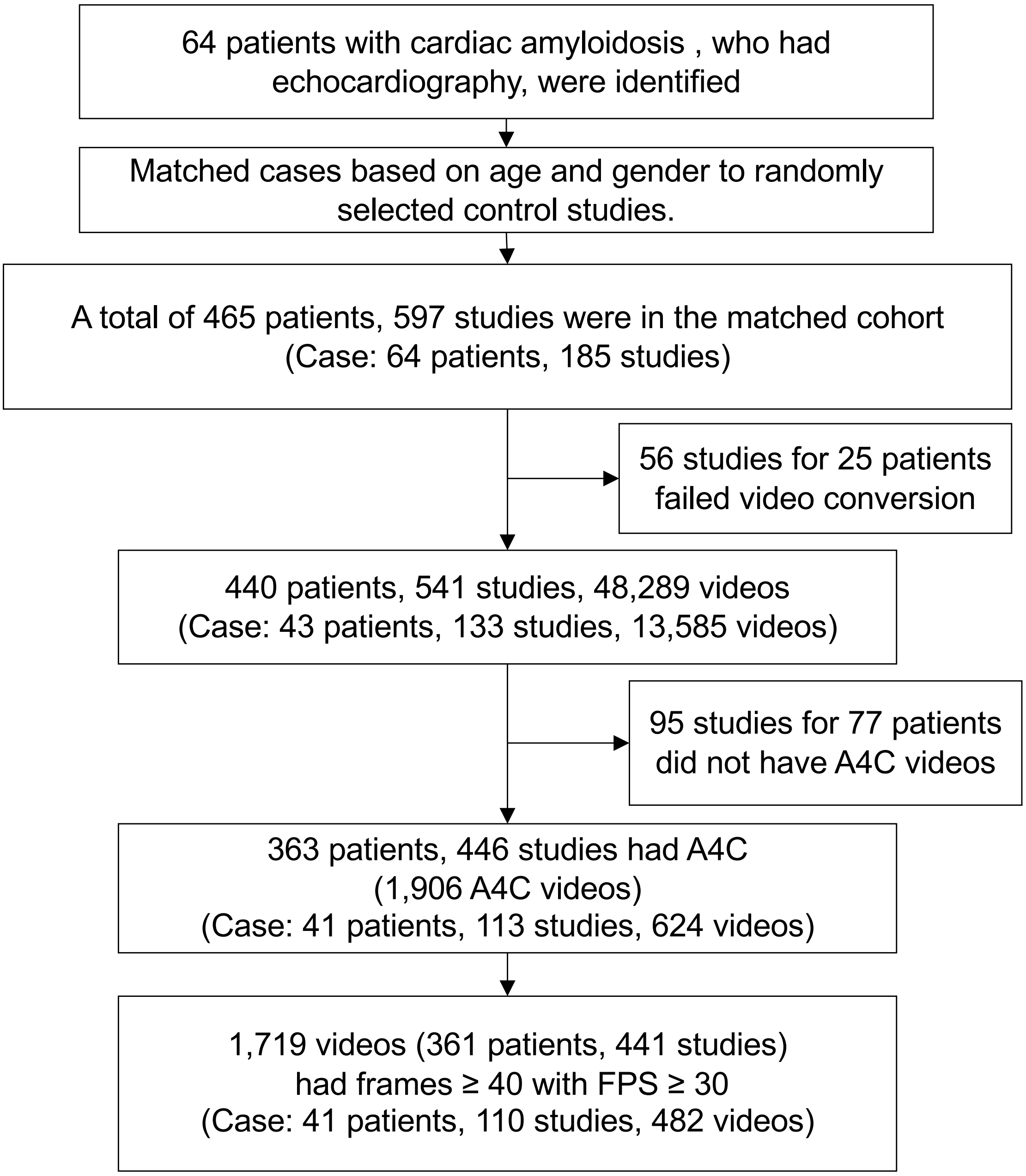

Supplementary Figure 11. Cohort selection procedure for the MGH echocardiography cohort.

MGH: Massachusetts General Hospital, FPS: frames per second, A4C: apical 4 chamber view.

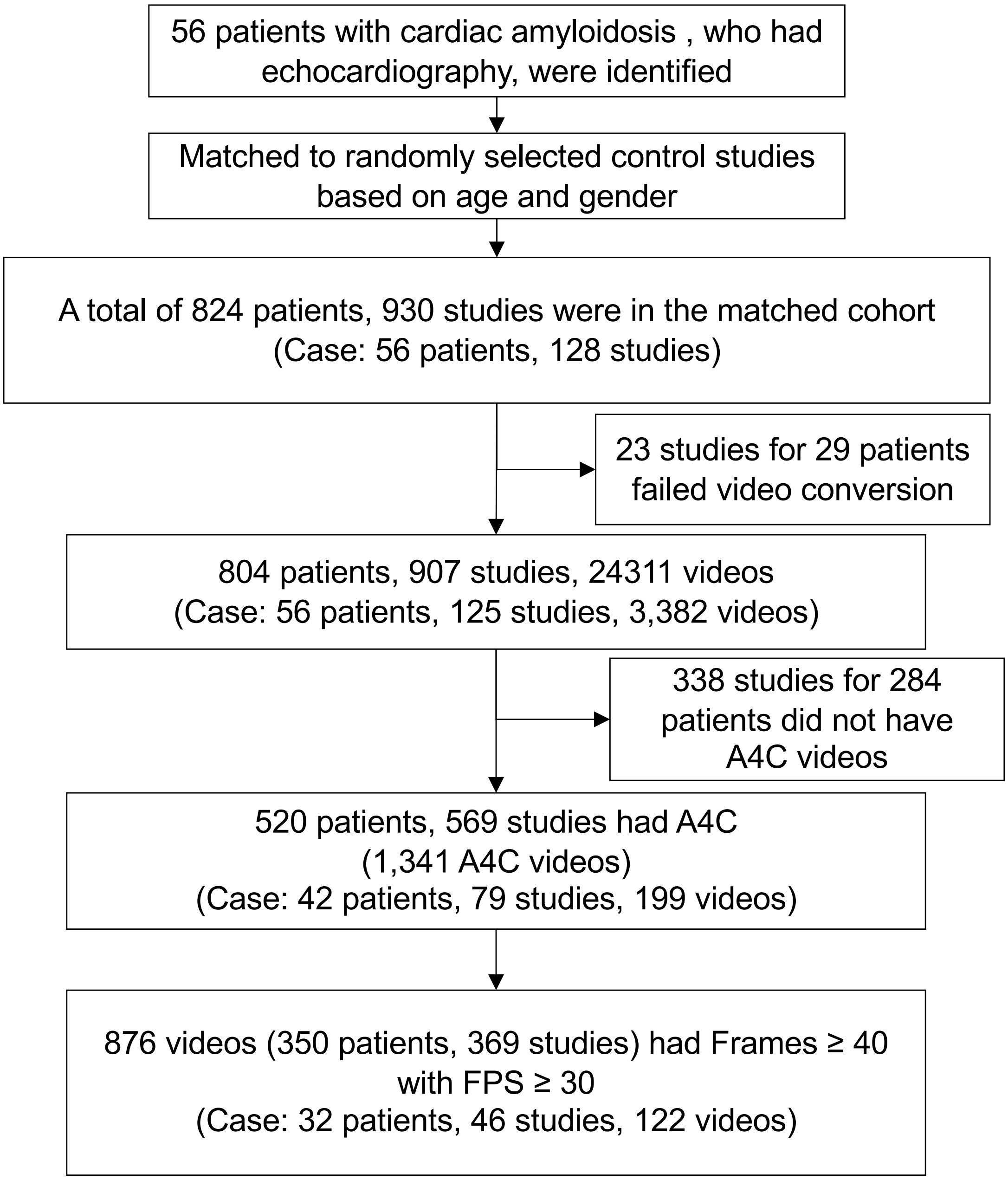

Supplementary Figure 12. Cohort selection procedure for the UCSF echocardiography cohort.

UCSF: University of California San Francisco, FPS: frames per second, A4C: apical 4 chamber view.

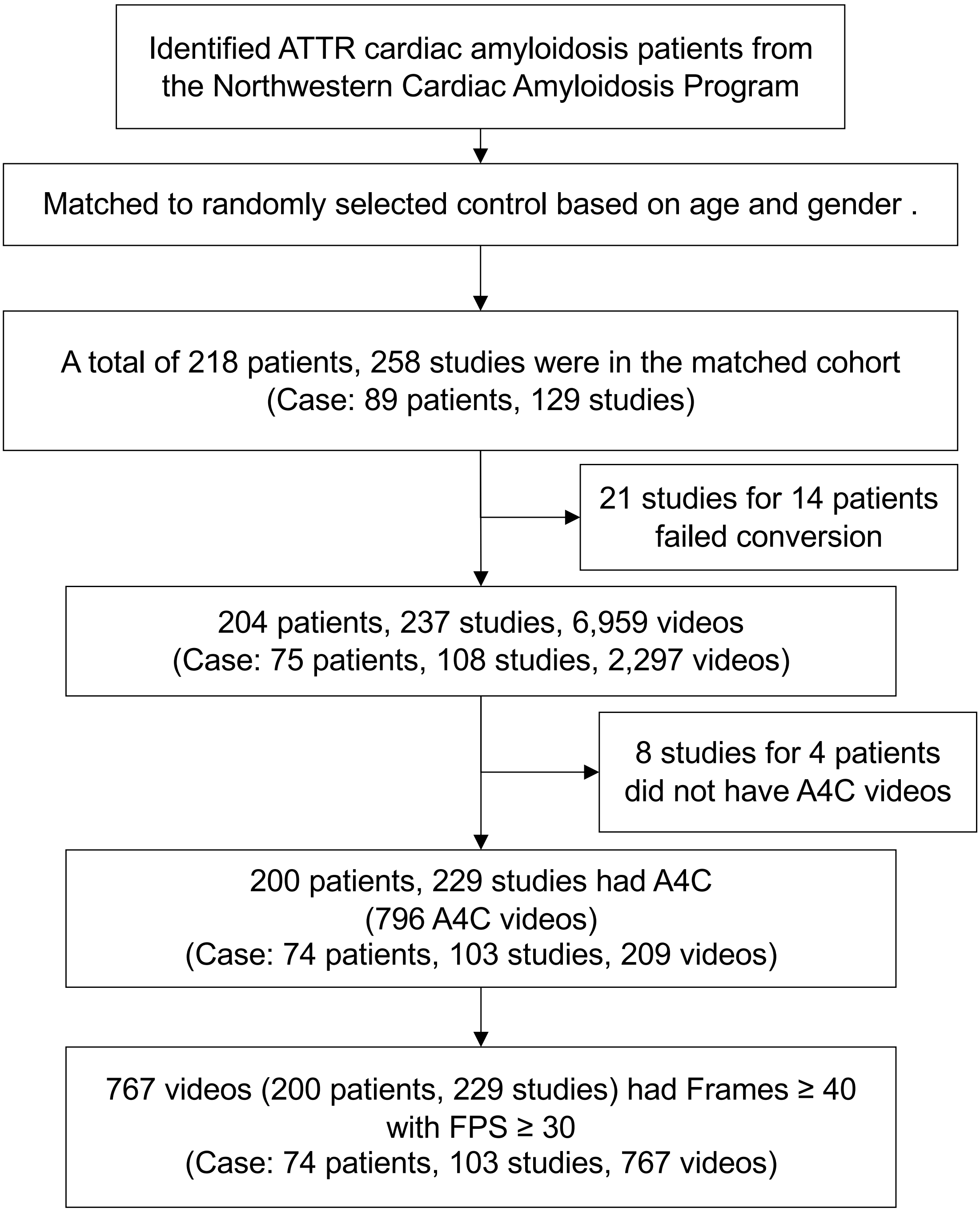

Supplementary Figure 13. Cohort selection procedure for the NW echocardiography cohort.

NW: Northwestern University, ATTR: amyloid transthyretin, FPS: frames per second, A4C: apical 4 chamber view.

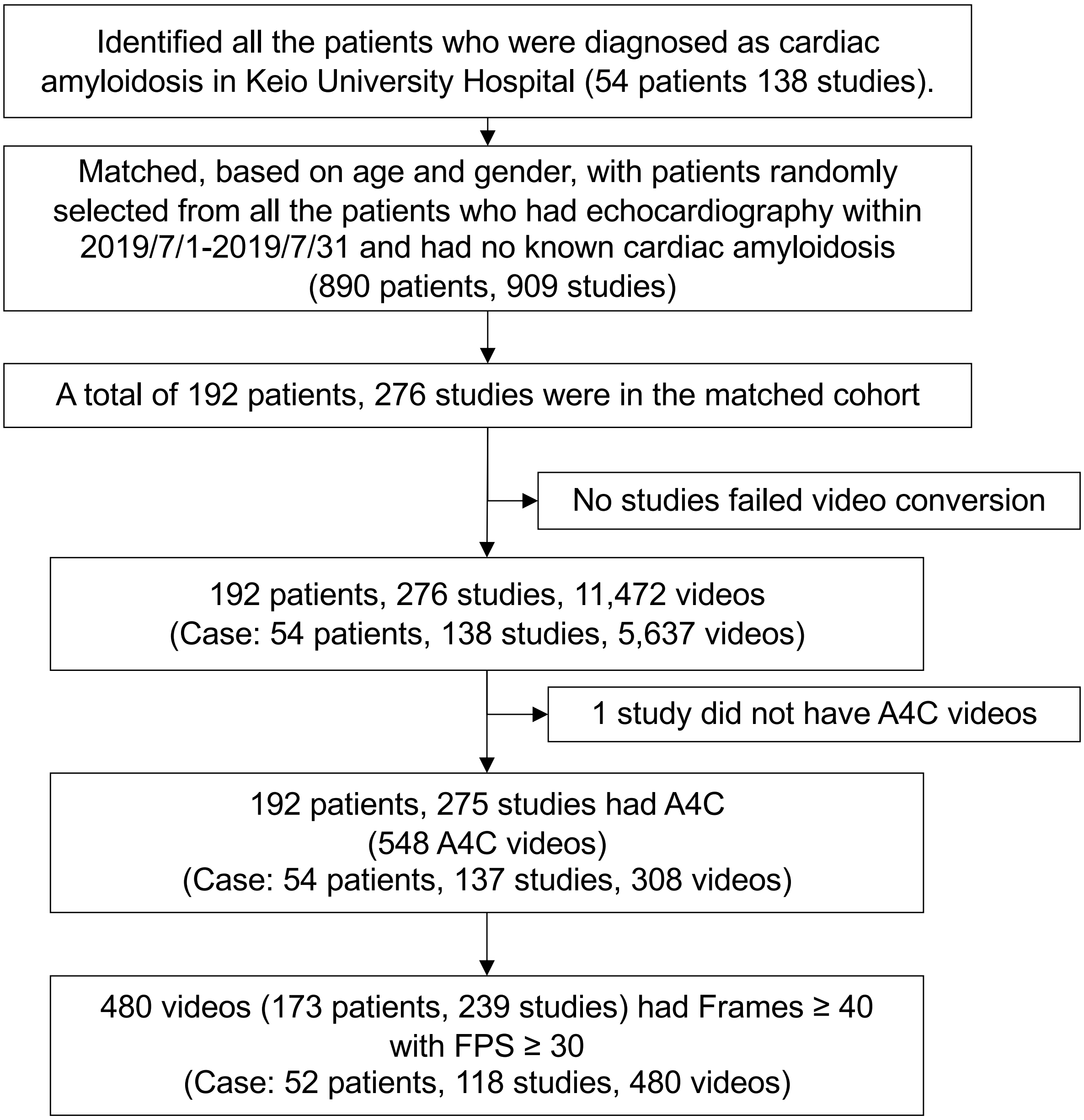

Supplementary Figure 14. Cohort selection procedure for the Keio echocardiography cohort.

Keio: Keio University, FPS: frames per second, A4C: apical 4 chamber view.

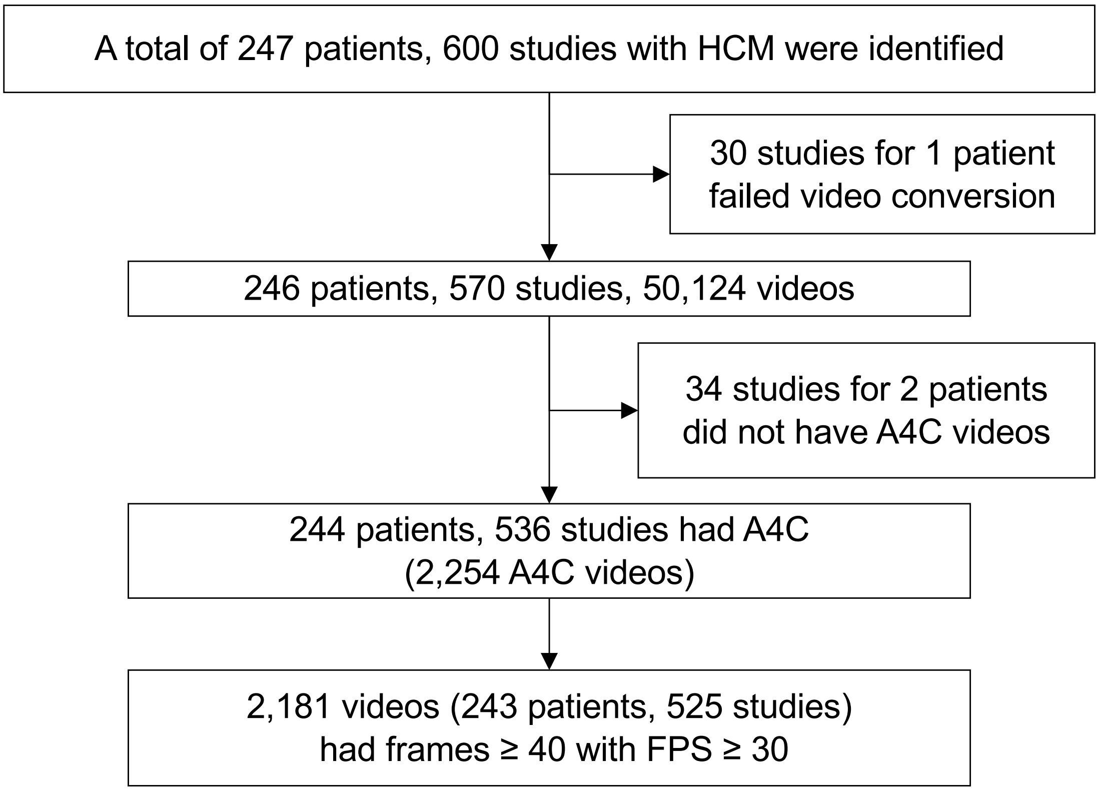

Supplementary Figure 15. Cohort selection procedure for the MGH echocardiography HCM comparison group.

MGH: Massachusetts General Hospital, HCM: hypertrophic cardiomyopathy, FPS: frames per second, A4C: apical 4 chamber view.

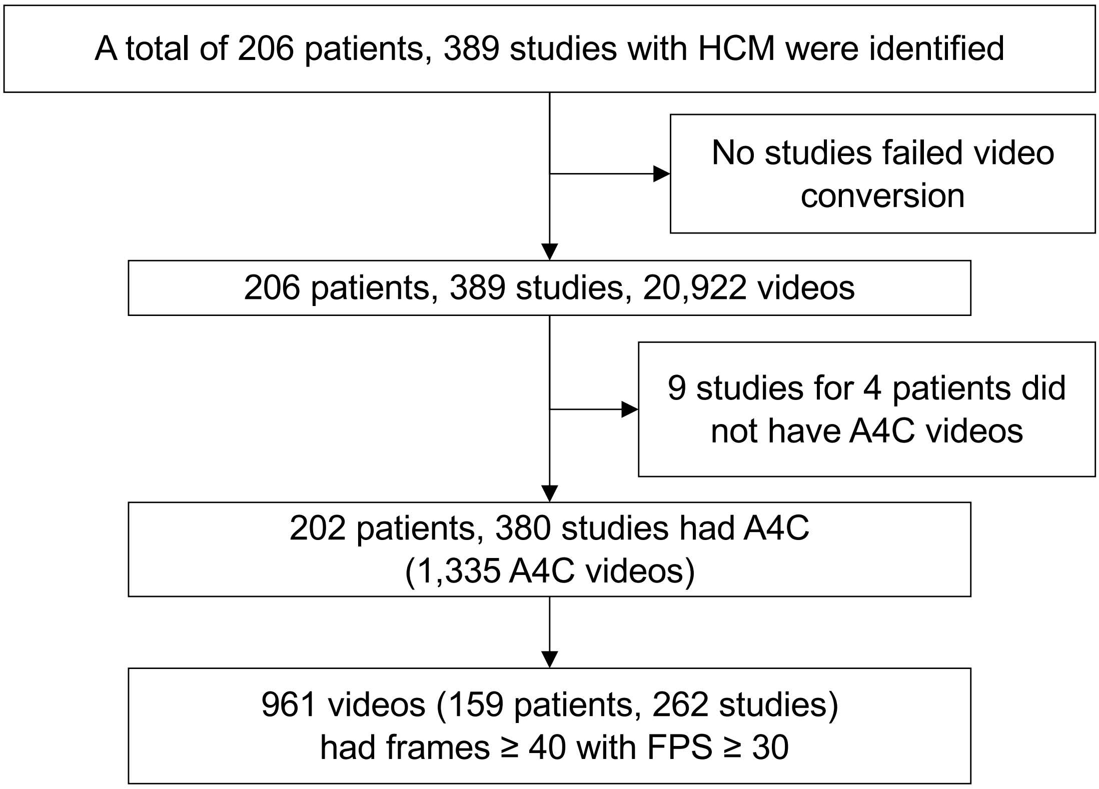

Supplementary Figure 16. Cohort selection procedure for the UCSF echocardiography HCM comparison group.

UCSF: University of California San Francisco, HCM: hypertrophic cardiomyopathy, FPS: frames per second, A4C: apical 4 chamber view.

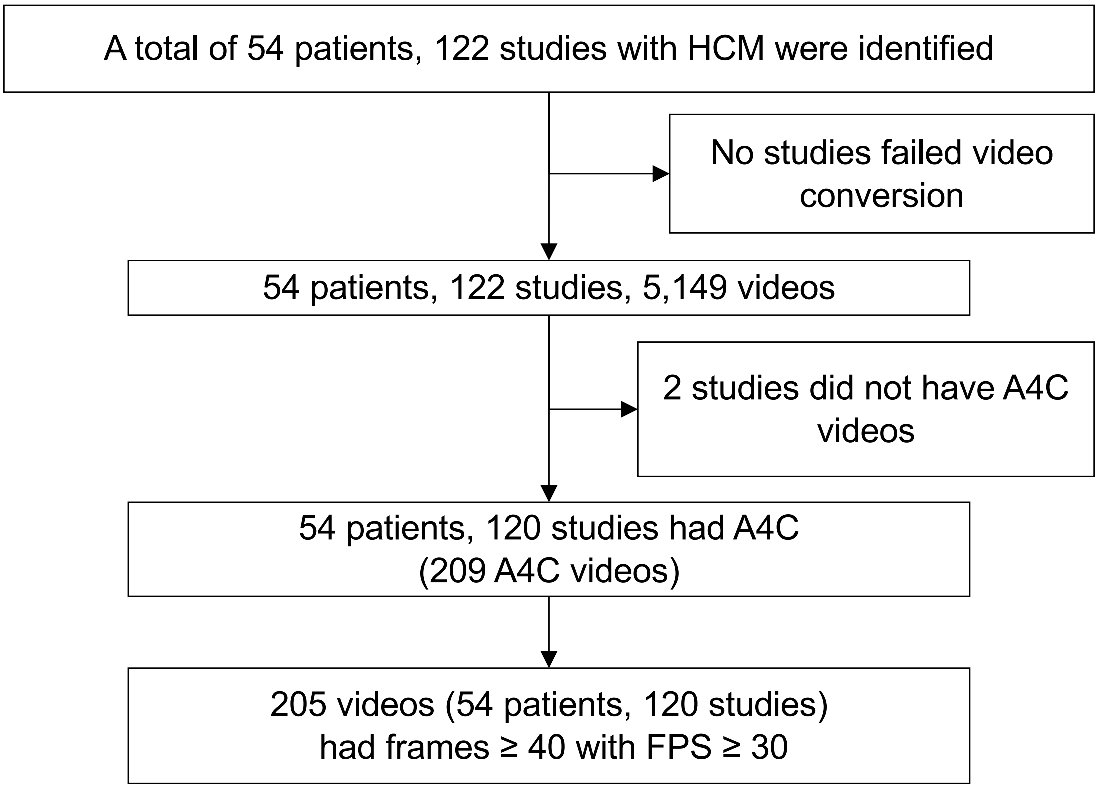

Supplementary Figure 17. Cohort selection procedure for the Keio echocardiography HCM comparison group.

Keio: Keio University, HCM: hypertrophic cardiomyopathy, FPS: frames per second, A4C: apical 4 chamber view.

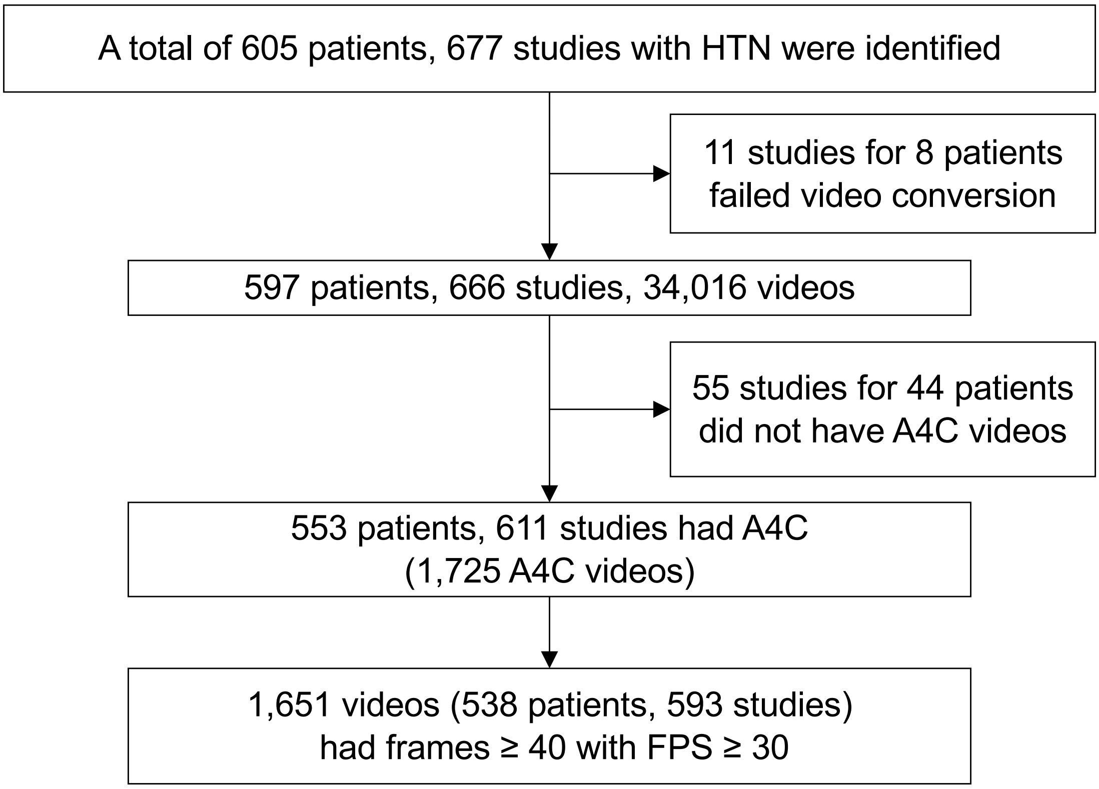

Supplementary Figure 18. Cohort selection procedure for the BWH echocardiography hypertension comparison group.

BWH: Brigham and Women's Hospital, HTN: hypertension, FPS: frames per second, A4C: apical 4 chamber view.

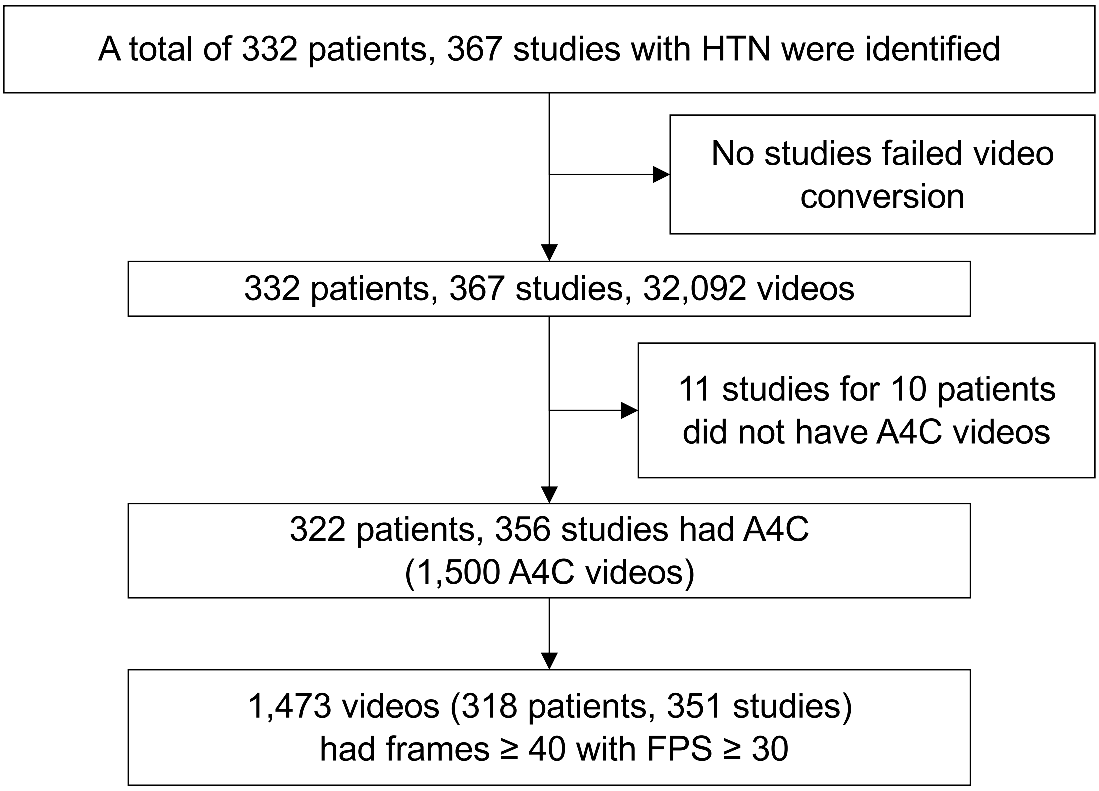

Supplementary Figure 19. Cohort selection procedure for the MGH echocardiography hypertension comparison group.

MGH: Massachusetts General Hospital, HTN: hypertension, FPS: frames per second, A4C: apical 4 chamber view.

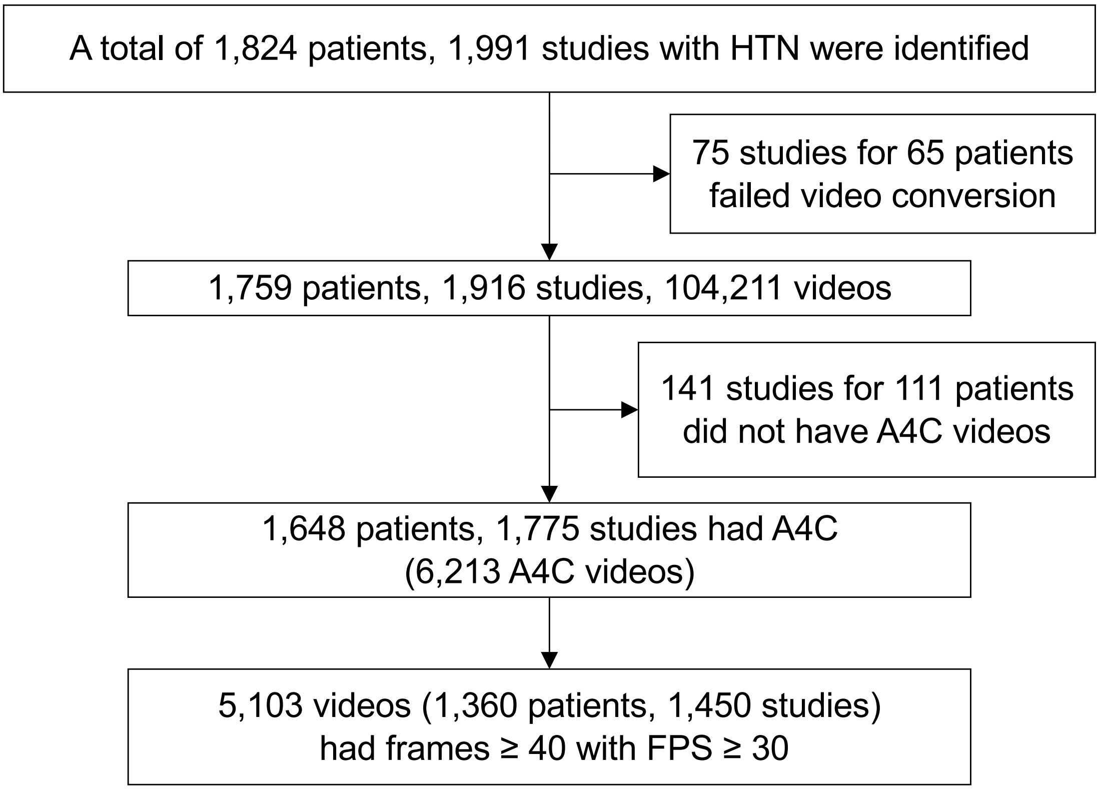

Supplementary Figure 20. Cohort selection procedure for the UCSF echocardiography hypertension comparison group.

UCSF: University of California San Francisco, HTN: hypertension, FPS: frames per second, A4C: apical 4 chamber view.

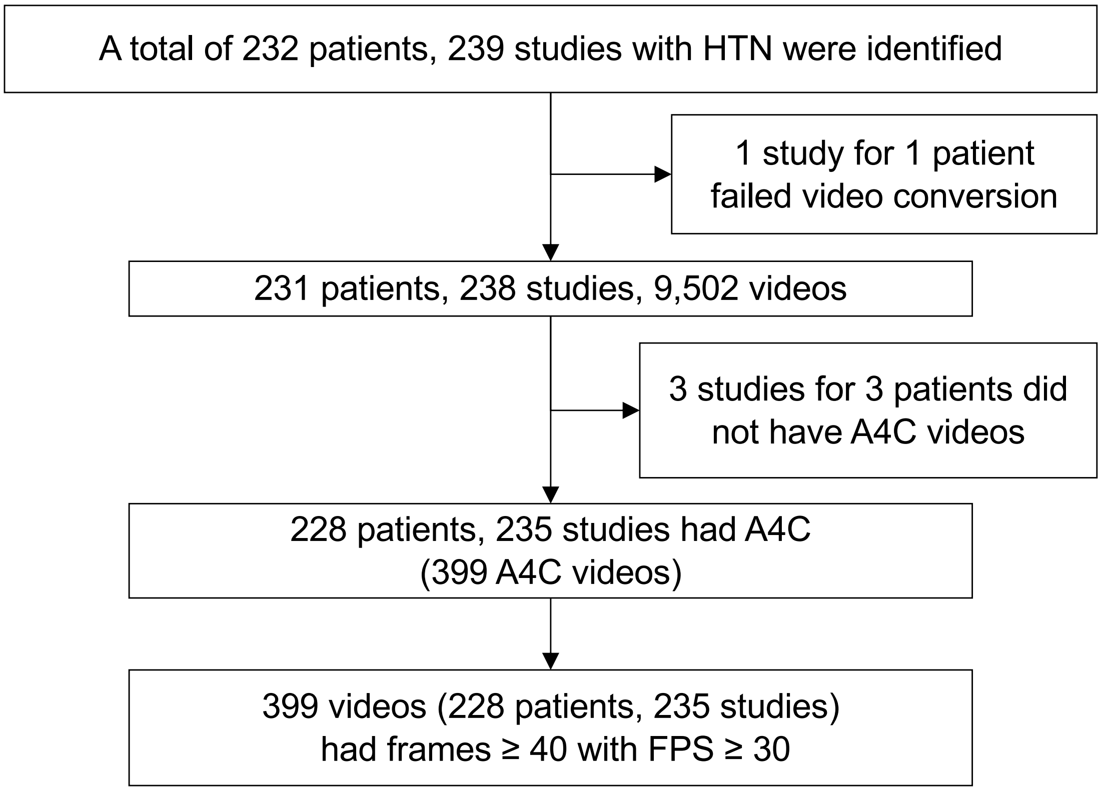

Supplementary Figure 21. Cohort selection procedure for the Keio echocardiography hypertension comparison group.

Keio: Keio University, HTN: hypertension, FPS: frames per second, A4C: apical 4 chamber view.

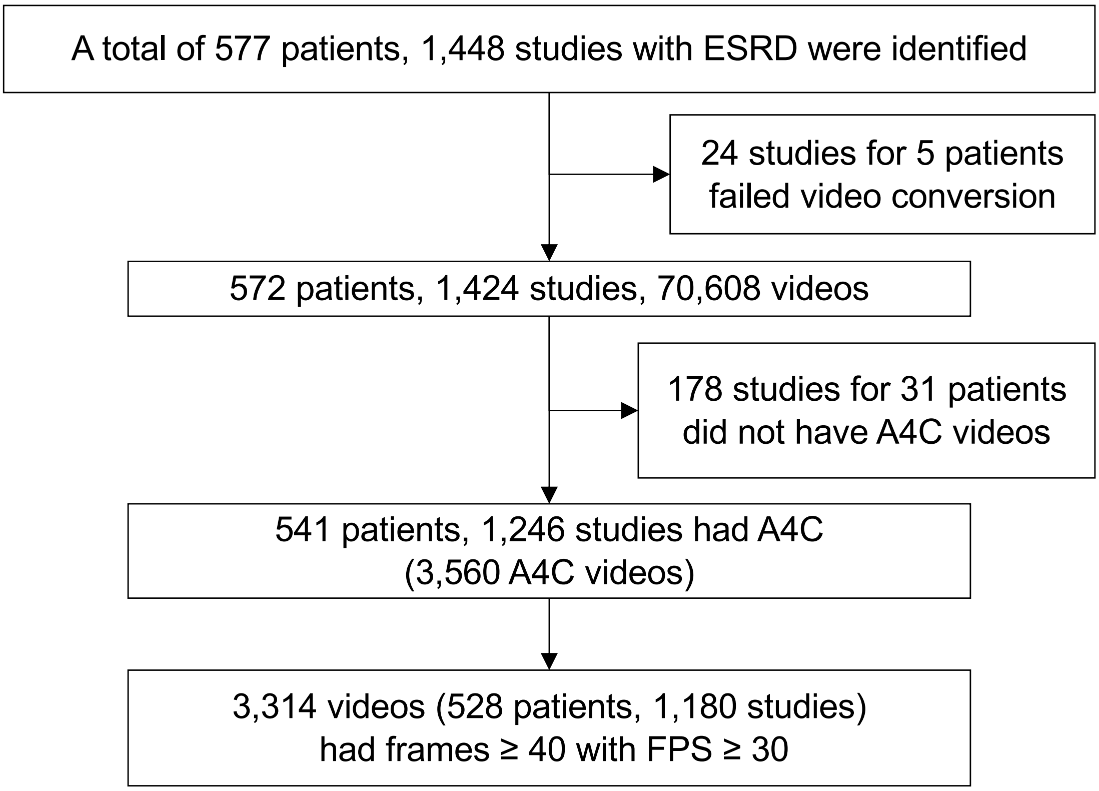

Supplementary Figure 22. Cohort selection procedure for the BWH echocardiography end-stage renal disease controls.

BWH: Brigham and Women's Hospital, ERSD: end-stage renal disease, FPS: frames per second, A4C: apical 4 chamber view.

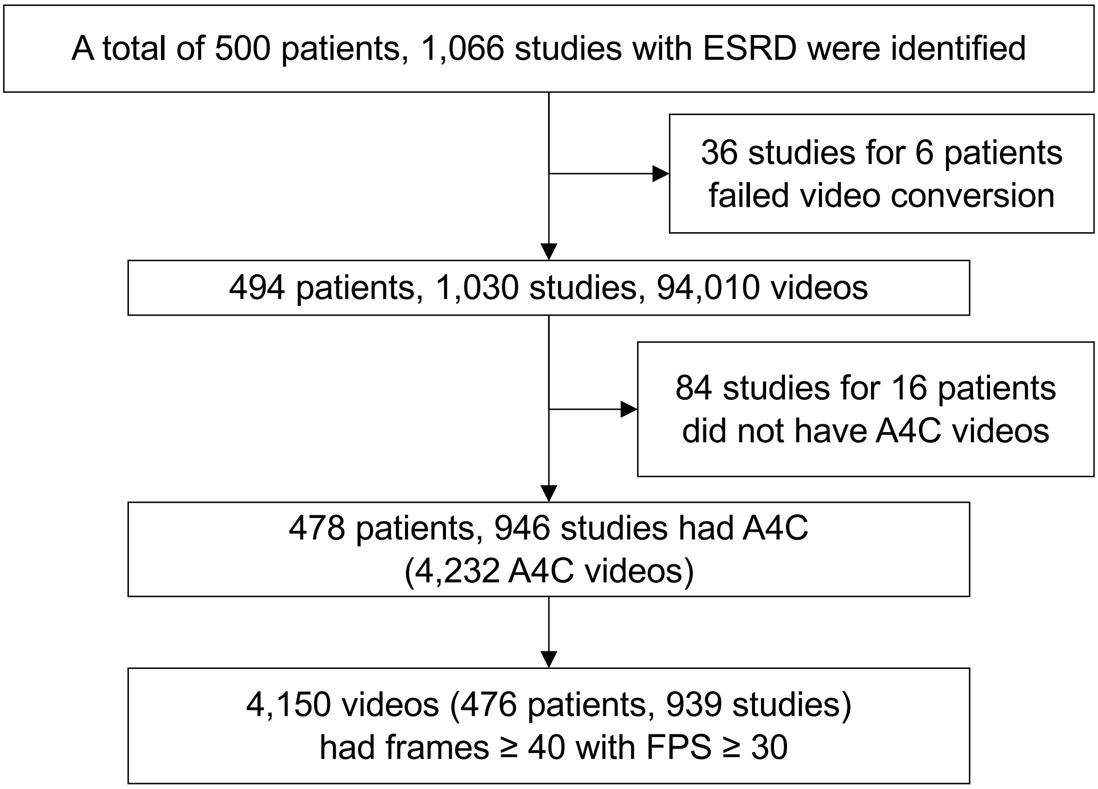

Supplementary Figure 23. Cohort selection procedure for the MGH echocardiography end-stage renal disease controls.

MGH: Massachusetts General Hospital, ERSD: end-stage renal disease, FPS: frames per second, A4C: apical 4 chamber view.

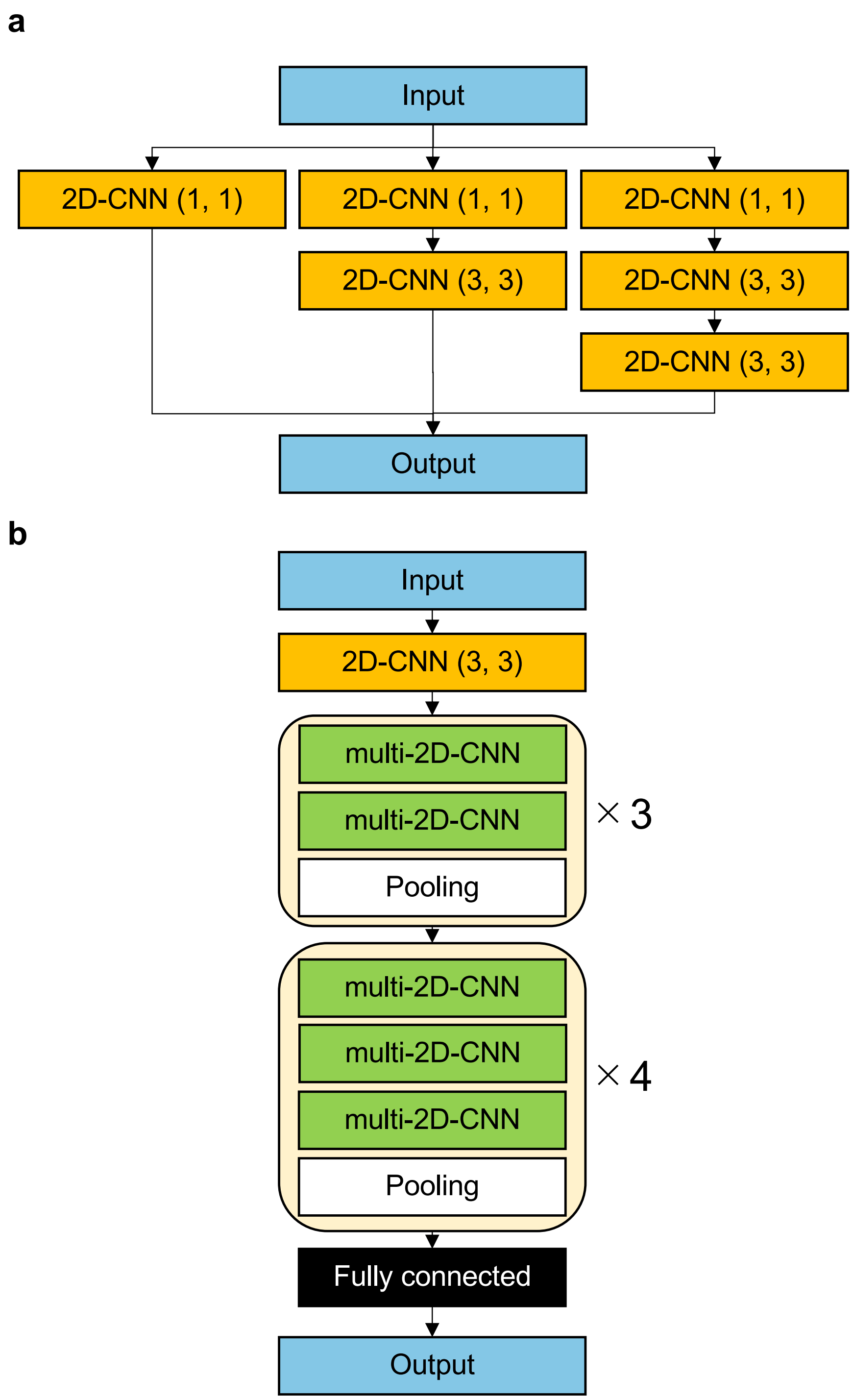

Supplementary Figure 24. Schematic drawing of the network structure of ECG model.

(A) Structure of the multi-2D-CNN module and (B) the full network structure of ECG model. ECG: electrocardiogram, CNN: convolutional neural network.

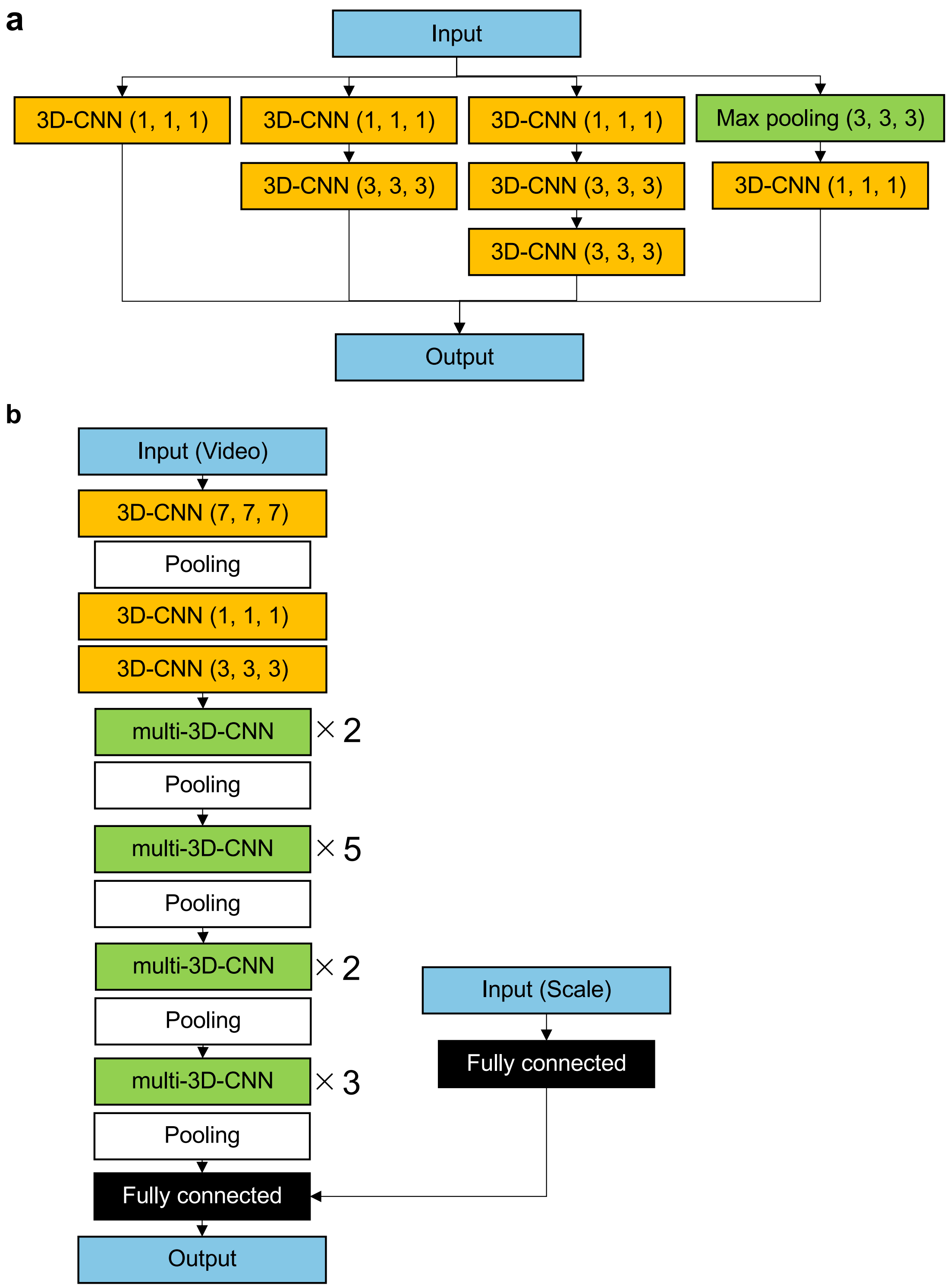

Supplementary Figure 25. Schematic drawing of the network structure of echocardiography model.

(A) Structure of the multi-3D-CNN module and (B) the full network structure of echocardiography model. CNN: convolutional neural network.

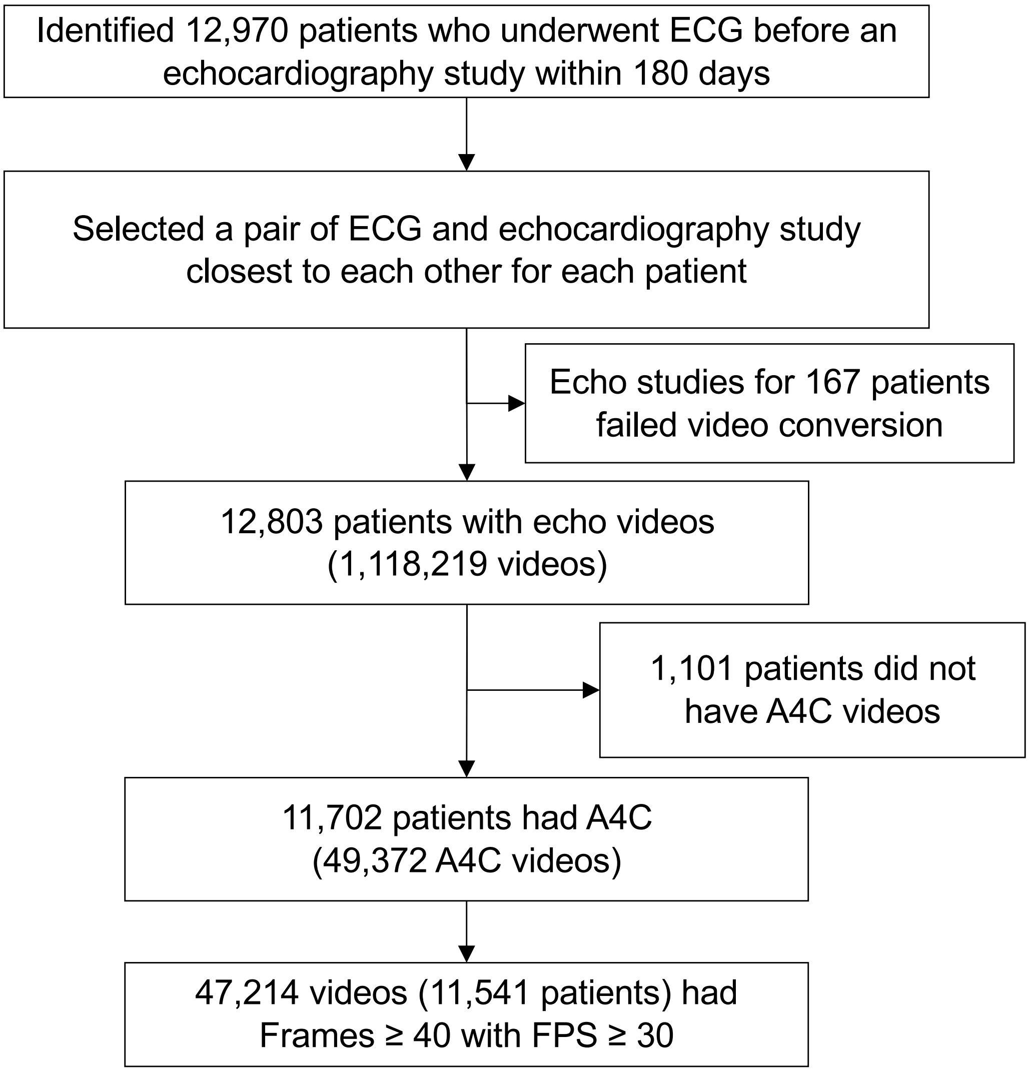

Supplementary Figure 26. Cohort selection procedure for the MGH surveillance cohort.

MGH: Massachusetts General Hospital, ECG: electrocardiogram, FPS: frames per second, A4C: apical 4 chamber view.

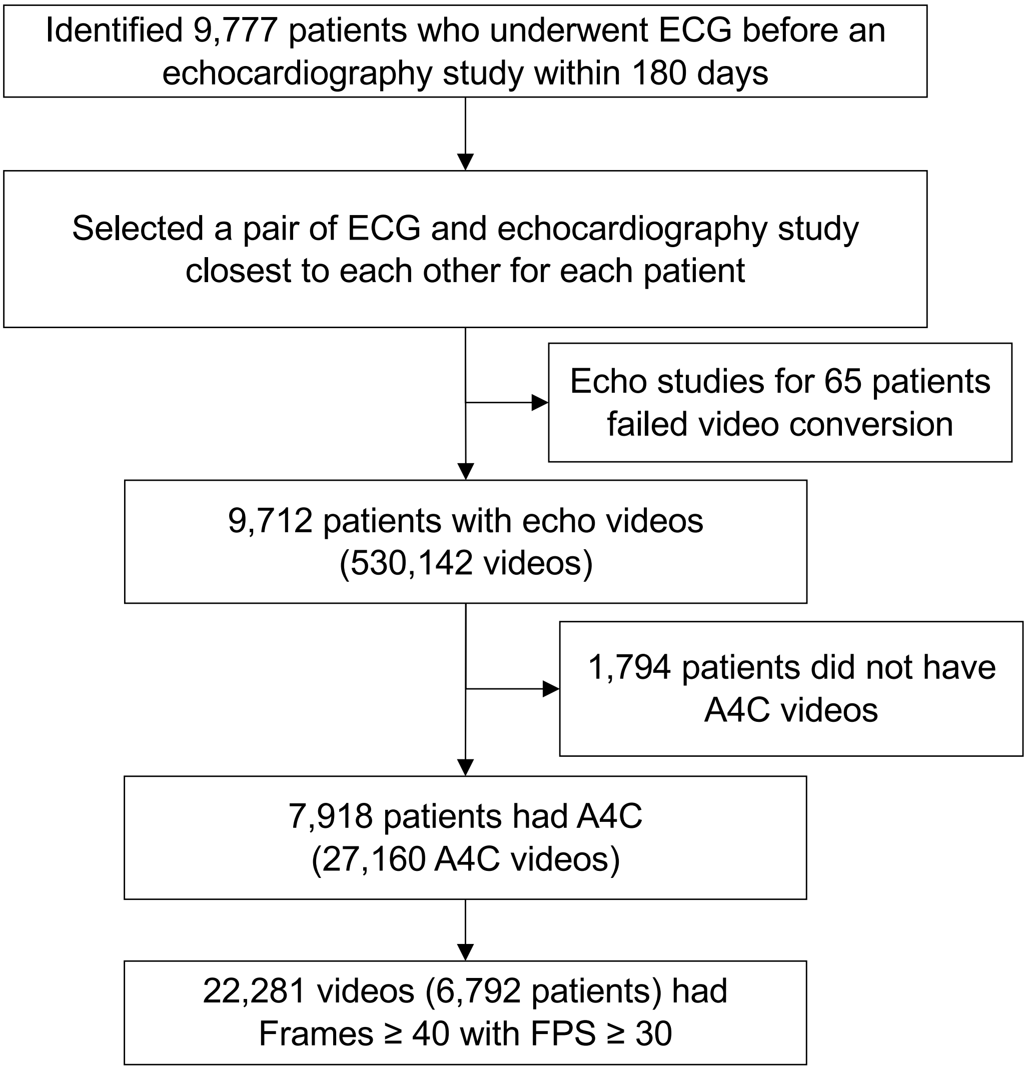

Supplementary Figure 27. Cohort selection procedure for the UCSF surveillance cohort.

UCSF: University of California San Francisco, ECG: electrocardiogram, FPS: frames per second, A4C: apical 4 chamber view.
