# Supplementary material for: Artificial intelligence-enabled fully automated detection of cardiac amyloidosis using electrocardiograms and echocardiograms
Source: Nat Commun. 2021 May 11;12:2726. doi: 10.1038/s41467-021-22877-8 (PMC8113484; doi:10.1038/s41467-021-22877-8)
Supplement: Supplementary file 2 — Reporting Summary [file 41467_2021_22877_MOESM2_ESM.pdf]

nature research

Corresponding author(s): Rahul C. Deo

Last updated by author(s): Nov 3 2020

Reporting Summary

Nature Research wishes to improve the reproducibility of the work that we publish. This form provides structure for consistency and transparency in reporting. For further information on Nature Research policies, see our Editorial Policies and the Editorial Policy Checklist.

Statistics

For all statistical analyses, confirm that the following items are present in the figure legend, table legend, main text, or Methods section.

n/a	Confirmed	
☐	☒	The exact sample size (*n*) for each experimental group/condition, given as a discrete number and unit of measurement
☐	☒	A statement on whether measurements were taken from distinct samples or whether the same sample was measured repeatedly
☐	☒	The statistical test(s) used AND whether they are one- or two-sided<br>*Only common tests should be described solely by name; describe more complex techniques in the Methods section.*
☒	☐	A description of all covariates tested
☒	☐	A description of any assumptions or corrections, such as tests of normality and adjustment for multiple comparisons
☐	☒	A full description of the statistical parameters including central tendency (e.g. means) or other basic estimates (e.g. regression coefficient) AND variation (e.g. standard deviation) or associated estimates of uncertainty (e.g. confidence intervals)
☒	☐	For null hypothesis testing, the test statistic (e.g. *F*, *t*, *r*) with confidence intervals, effect sizes, degrees of freedom and *P* value noted<br>*Give P values as exact values whenever suitable.*
☒	☐	For Bayesian analysis, information on the choice of priors and Markov chain Monte Carlo settings
☒	☐	For hierarchical and complex designs, identification of the appropriate level for tests and full reporting of outcomes
☒	☐	Estimates of effect sizes (e.g. Cohen's *d*, Pearson's *r*), indicating how they were calculated

Our web collection on statistics for biologists contains articles on many of the points above.

Software and code

Policy information about availability of computer code

Data collection	All data were collected using Python 3.7.3 and Numpy 1.19.2
Data analysis	All the models were trained with Keras 2.3.0 on a Tensorflow 1.14.0 backend. The ROC curves are plotted using the ggplot2 package (R 3.6.1) and the AUC-ROC, sensitivity, specificities, and 95% confidence intervals (using 2000 bootstrap samples) were calculated using pROC package (version 1.16.2). The precision-recall plots were made using the plotnine package (version 0.6.0) in Python 3.7.3.<br>Codes for running the model are available as supplementary materials attached to the manuscript. A web-based environment to run our model is available at http://onebraveideaml.org.

For manuscripts utilizing custom algorithms or software that are central to the research but not yet described in published literature, software must be made available to editors and reviewers. We strongly encourage code deposition in a community repository (e.g. GitHub). See the Nature Research guidelines for submitting code & software for further information.

Data

Policy information about availability of data

All manuscripts must include a data availability statement. This statement should provide the following information, where applicable:

- Accession codes, unique identifiers, or web links for publicly available datasets
- A list of figures that have associated raw data
- A description of any restrictions on data availability

The code for running the model is attached as supplementary file.
The raw data of patients can not be shared since they include personal information.
The model weights may also contain patient personal information and thus, could not be shared. We provide a web-interface to run our model and calculate predictions in http://onebraveideaml.org

Field-specific reporting

Please select the one below that is the best fit for your research. If you are not sure, read the appropriate sections before making your selection.

- [x] Life sciences
- [ ] Behavioural & social sciences
- [ ] Ecological, evolutionary & environmental sciences

For a reference copy of the document with all sections, see nature.com/documents/nr-reporting-summary-flat.pdf

Life sciences study design

All studies must disclose on these points even when the disclosure is negative.

Sample size	N/A (not a hypothesis testing study)
Data exclusions	Echo study that failed conversion, that did not have A4C video were excluded.<br>Echo videos with low frame rate < 30 fps or short length < less than 1.3 sec were excluded.
Replication	The study results were replicated 3 and 5 academic medical centers from USA and Japan.
Randomization	The patients were randomly selected using the random number generator implemented in Python, numpy and tensorflow.
Blinding	In the "Echocardiography model comparison with expert cardiologist interpretation" the experts were blinded to model outcomes and clinical measurements.

Reporting for specific materials, systems and methods

We require information from authors about some types of materials, experimental systems and methods used in many studies. Here, indicate whether each material, system or method listed is relevant to your study. If you are not sure if a list item applies to your research, read the appropriate section before selecting a response.

Materials & experimental systems

n/a	Involved in the study	
x		Antibodies
x		Eukaryotic cell lines
x		Palaeontology and archaeology
x		Animals and other organisms
	x	Human research participants
x		Clinical data
x		Dual use research of concern

Methods

n/a	Involved in the study	
x		ChIP-seq
x		Flow cytometry
x		MRI-based neuroimaging

Human research participants

Policy information about studies involving human research participants

Population characteristics	The population characteristics are reported as tables in the manuscript
Recruitment	The patient recruitment methods are fully explained in the manuscript.
Ethics oversight	Partners health care, University of California San Fransisco, North Western University, Keio University

Note that full information on the approval of the study protocol must also be provided in the manuscript.
